# Supplementary material for: Enhancer RNAs stimulate Pol II pause release by harnessing multivalent interactions to NELF
Source: Nat Commun. 2022 May 4;13:2429. doi: 10.1038/s41467-022-29934-w (PMC9068813; doi:10.1038/s41467-022-29934-w)
Supplement: Supplementary file 1 — Supplementary Information [file 41467_2022_29934_MOESM1_ESM.pdf]

# Supplementary Information

## Enhancer RNAs stimulate Pol II pause release by harnessing multivalent interactions to NELF

Vladyslava Gorbovytska[1,*], Seung-Kyoon Kim[2,3,*], Filiz Kuybu[1], Michael Götze[4], Dahun Um[2], Keunsoo Kang[5], Andreas Pittroff[1], Theresia Brennecke[1], Lisa-Marie Schneider[1], Alexander Leitner[4], Tae-Kyung Kim[2,6,§], and Claus-D. Kuhn[1,§]

[1] RNA Biochemistry, University of Bayreuth, Universitätsstrasse 30, 95447 Bayreuth, Germany

[2] Department of Life Sciences, Pohang University of Science and Technology (POSTECH), Pohang, Gyeongbuk, 37673, Republic of Korea

[3] Department of Convergent Bioscience and Informatics, College of Bioscience and Biotechnology, Chungnam National University, Daejeon, 34134, Republic of Korea

[4] Department of Biology, Institute of Molecular Systems Biology, ETH Zurich, 8093 Zurich, Switzerland

[5] Department of Microbiology, Dankook University, Cheonan, 31116, Republic of Korea

[6] Institute of Convergence Science, Yonsei University, Seoul, 03722, Republic of Korea

[*] These authors contributed equally to this work.

[§] Correspondence should be addressed to: T.-K. Kim: tkkim@postech.ac.kr

C.-D. Kuhn: claus.kuhn@uni-bayreuth.de

**Supplementary Information includes:**

11 Supplementary Figures
1 Supplementary Reference list

# Supplementary Fig. 1

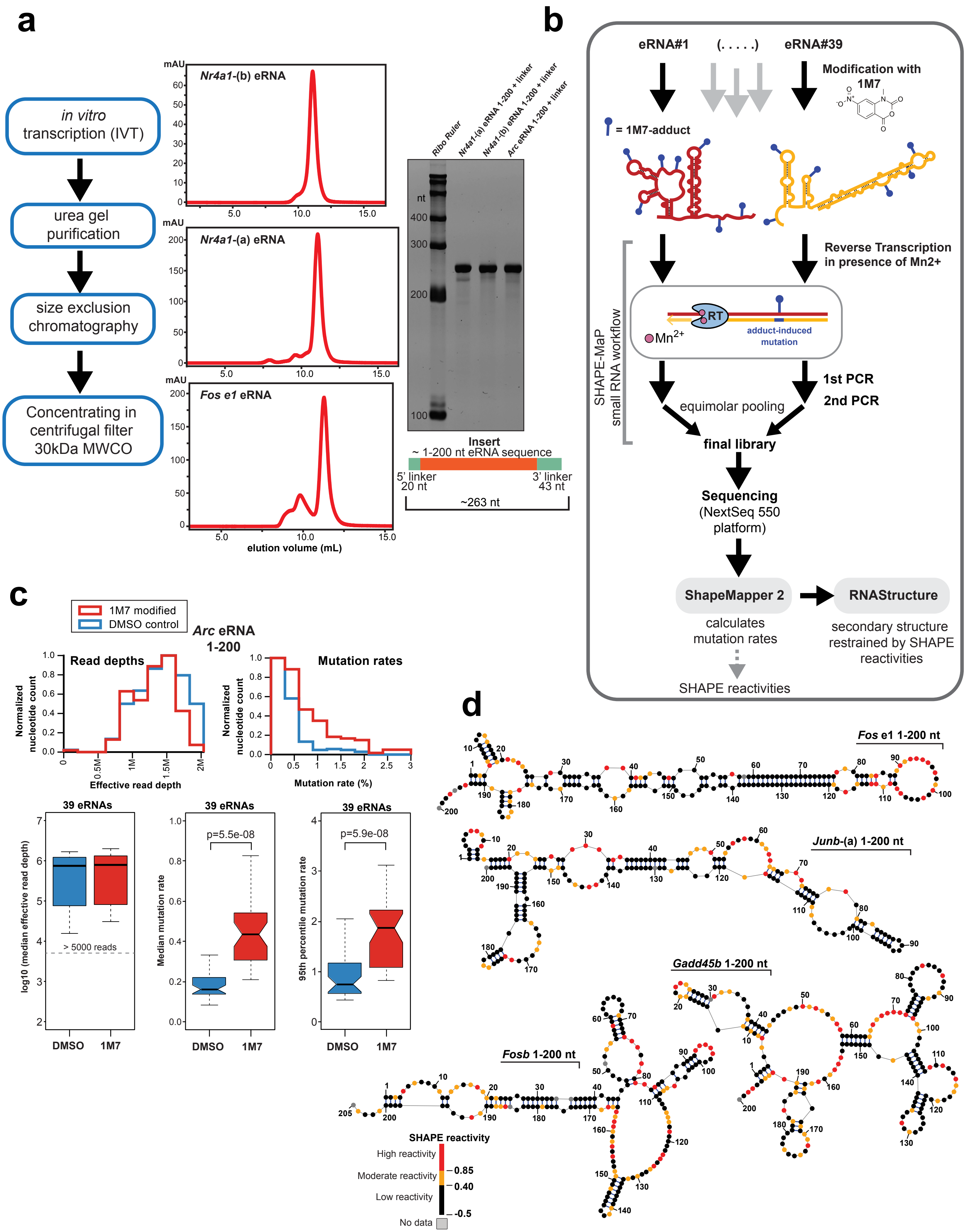

**Supplementary Fig. 1 | SHAPE-MaP workflow and mapping statistics. a,** Workflow of eRNA production for SHAPE-MaP (left panel). Representative gel filtration chromatograms of three eRNAs (middle panel) and an analytical urea-PAGE of purified *Nr4a1*-(a), *Nr4a1*-(b) and *Arc* eRNAs (right panel). SHAPE-MaP eRNAs comprise the first 200 nucleotides (1-200) of each eRNA flanked by independently folding 5' and 3' structure cassettes, yielding a total length of 263 nt for the produced RNAs (see scheme below the gel and Methods for details). **b,** Workflow of SHAPE-MaP library construction and data analysis after sequencing. **c,** SHAPE-MaP statistics indicate a successful experiment. Read depth and mutation rate histograms for *Arc* eRNA are shown as an example (top panel, output from *Shapemapper2*). The bottom panel displays a boxplot of the median effective read depth (left plot) for all 39 eRNAs in the 1M7-modified and DMSO control samples (n=39). All samples pass recommended quality criteria of > 5000 reads[1]. The additional boxplots (middle, right plot) display the median and 95$^{th}$ percentile mutation rates for the DMSO control and 1M7-modified samples (n=39). The obtained mutation rates for the 1M7 treatment lie significantly above the background mutation rates of the DMSO-control, as determined by the nonparametric, two-sided, Mann-Whitney-Wilcoxon test for paired samples (median mutation rates: p=7.7e-8; and 95$^{th}$ perc. mutation rates: p=5.9e-8). See legend of Figure 1d for definition of the box-and-whisker-plot. **d,** Secondary structures based on the SHAPE-MaP data, as modeled by the MaxExpect algorithm of *RNAStructure*[2] for selected eRNAs (*Fos* e1, *Junb* variant (a), *Gadd45b*, *Fosb*) referring to immediate early genes. Nucleotides are shown as circles and are colored according to their SHAPE reactivity (see legend below). Source data for (a and c) are provided as a Source Data file.

# Supplementary Fig. 2

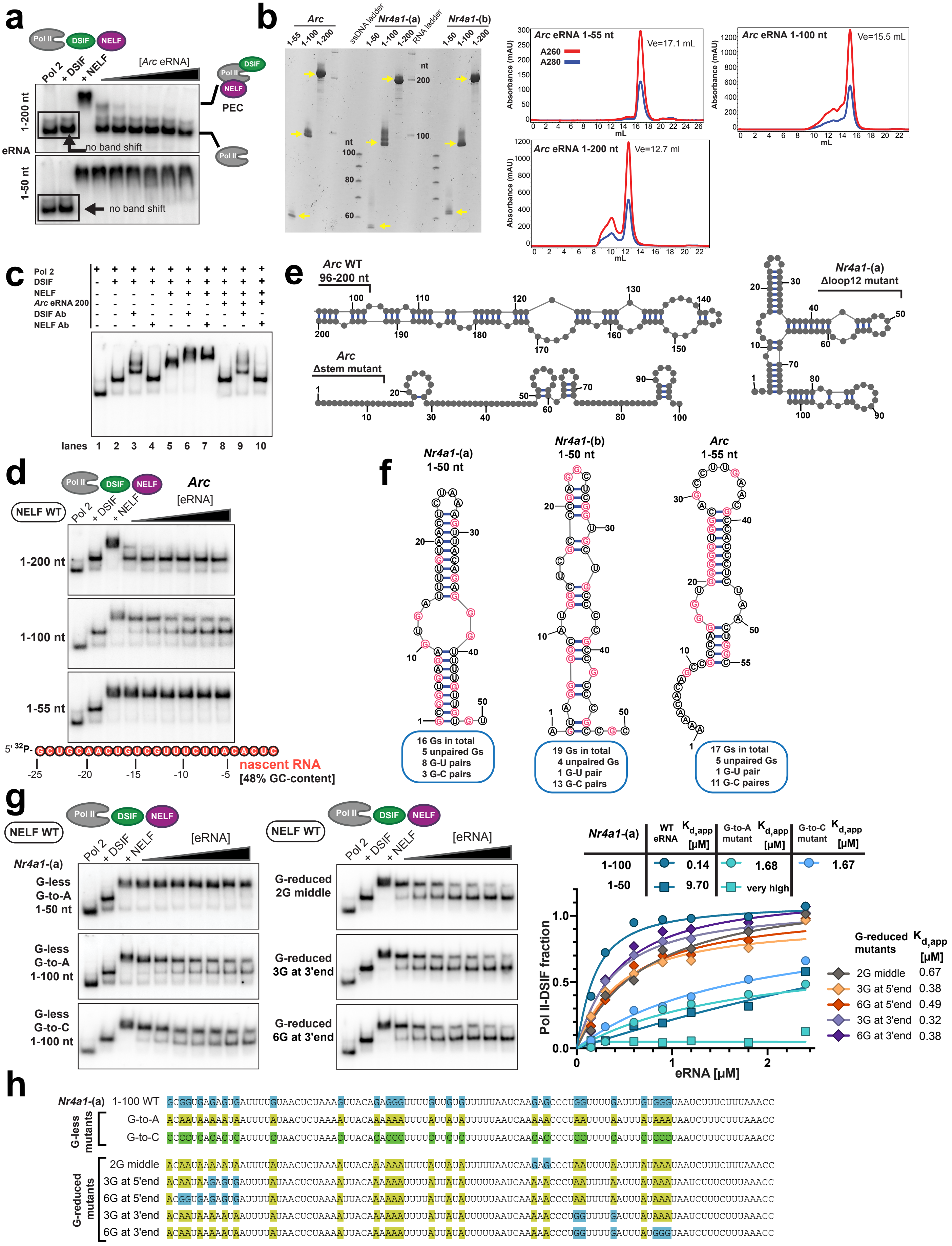

**Supplementary Fig. 2 | EMSA experiments demonstrate that eRNAs trigger NELF release from the paused elongation complex (PEC). a,** EMSA performed on a transcription bubble with a shorter, 15 nt long, nascent RNA (*versus* 25 nt long in Fig. 2b-d). Addition of DSIF does not lead to a shift of the mobility of the initial band that contains Pol II bound to the transcription bubble. Only utilization of a longer nascent RNA (25 nt; see main Fig. 2b-d) leads to a visible shift upon DSIF addition, which confirms the binding of DSIF to Pol II. **b,** Quality assessment of *in vitro* produced eRNAs that were used for functional assays. An analytical urea-PAGE for *Arc*, *Nr4a1*-(a) and -(b) fragments (1-50, 1-100 and 1-200) is shown. The right panel displays representative gel filtration (Superdex 200 Increase 10/300 GL column) chromatograms for the three *Arc* eRNA fragments. **c,** An EMSA supershift assay confirms the identity of the bands in the EMSA experiments (shown in Fig. 2, 3, Supplementary Fig. 2,3). To that end anti-Strep and anti-NELF-E antibodies were used (SPT5, the large subunit of DSIF, carries a Strep-tag in our experimental setup). Only the Strep-tag antibody against DSIF (lane 9) and not the NELF-E antibody (lane 10) is able to shift the band after *Arc* 1-200 nt eRNA addition (lane 8), reporting that NELF was dissociated from Pol II while DSIF stayed upon eRNA addition. **d,** EMSA performed on a Pol II transcription bubble with an alternative nascent RNA exhibiting a higher GC content (48%) as compared to the standard nascent RNA (28% GC-content) used for all EMSA experiments. The effect of the differently sized *Arc* eRNA fragments on the "high GC-content PEC" equals the effect on the standard PEC assembly, confirming that the sequence and GC-content of the nascent RNA does have an impact on the eRNA-driven dissociation of NELF. **e,** Expected secondary structures of the wild type *Arc* eRNA (96-200) fragment (length = 104 nt; according to the SHAPE-MaP structure of the 1-200 fragment shown in Fig. 1c), the *Arc* eRNA Δstem mutant (length = 100 nt; predicted structure) and the *Nr4a1*-(a) Δloop 12 mutant (length = 102 nt; according to the SHAPE-MaP structure 1-200 shown in Fig. 1e, in which all prominent single stranded regions were deleted). Nucleotides are shown as gray circles. The structures refer to the EMSA shown in Fig. 2f. **f,** Secondary structures of *Nr4a1*-(a), -(b) 1-50 and *Arc* 1-55 nt fragment predicted with *RNAStructure*. The structure of *Arc* eRNA (1-55) is consistent with the structure within the 1-200 nt fragment determined by SHAPE-MaP. All Gs are highlighted in pink. The total G content between the fragments is similar (16-19 Gs). While most of the guanosines in *Nr4a1*-(b) and *Arc* are paired with cytidines, guanosines in *Nr4a1*-(a) are located in loop regions or G-U pairs. **g,** In the left panels EMSAs performed with *Nr4a1*-(a) 1-50, 1-100 G-less and 1-100 G-reduced fragments are shown. The right panel displays the quantification of NELF release for all G-mutants shown on the left, plus two additional mutants (corresponding sequences of all G-less and G-reduced mutants are shown

in subfigure **h**). The quantification of wild-type fragments is added to aid comparison (see Fig. 2c). In G-less fragments all guanosines were substituted with either adenosine (G-to-A mutant) or cytidine (G-to-C mutant). In the G-reduced fragments different number of guanosines (2, 3 or 6) were restored either at the 3' or 5' of the sequence (see subfigure **h;** restored Gs are highlighted in blue). **h**, Alignment of the RNA sequences corresponding to the wildtype sequence of *Nr4a1*-(a) and therefrom derived sequences of the G-less and G-reduced mutants (explained in subfigure **g**). Source data for (b,c,d and g).

# Supplementary Fig. 3

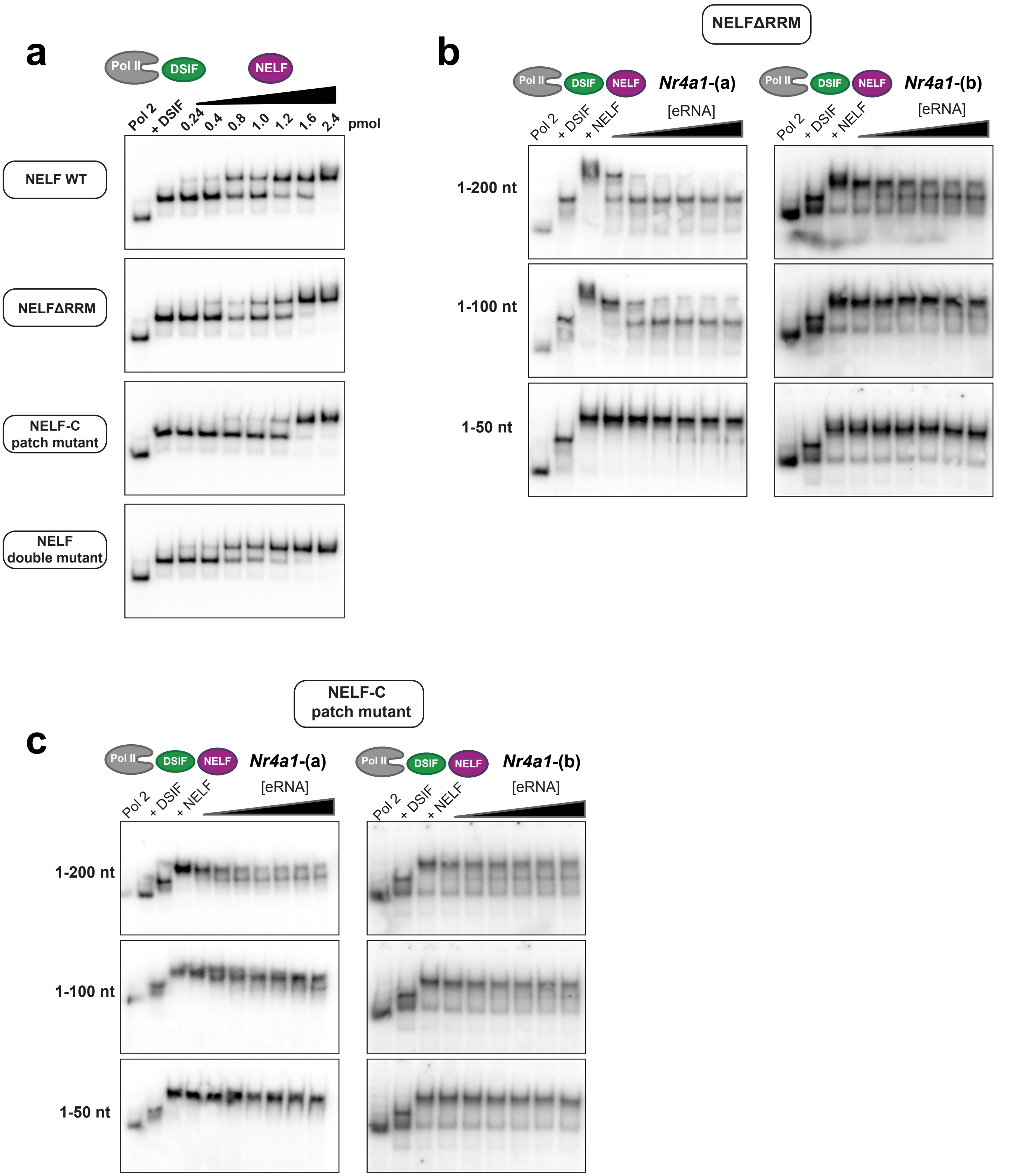

**Supplementary Fig. 3 | NELF mutants form the PEC as efficiently as wild-type NELF, however, their eRNA-driven detachment from Pol II is dramatically reduced. a,** An EMSA titration experiment of WT NELF and different NELF mutants (NELFΔRRM, NELF-C patch mutant and the double mutant) to preformed Pol II-DSIF complexes demonstrates that wild type and mutant NELF variants form the PEC comparably well. EMSAs performed with the fragments 1-50, 1-100 and 1-200 of *Nr4a1*-(a) and *Nr4a1*-(b) in the presence of either the NELFΔRRM mutant **(b)**, or the NELF patch mutant **(c)**. EMSAs were performed as described in Fig. 2b. Source data for (a-c) are provided as a Source Data file.

# Supplementary Fig. 4

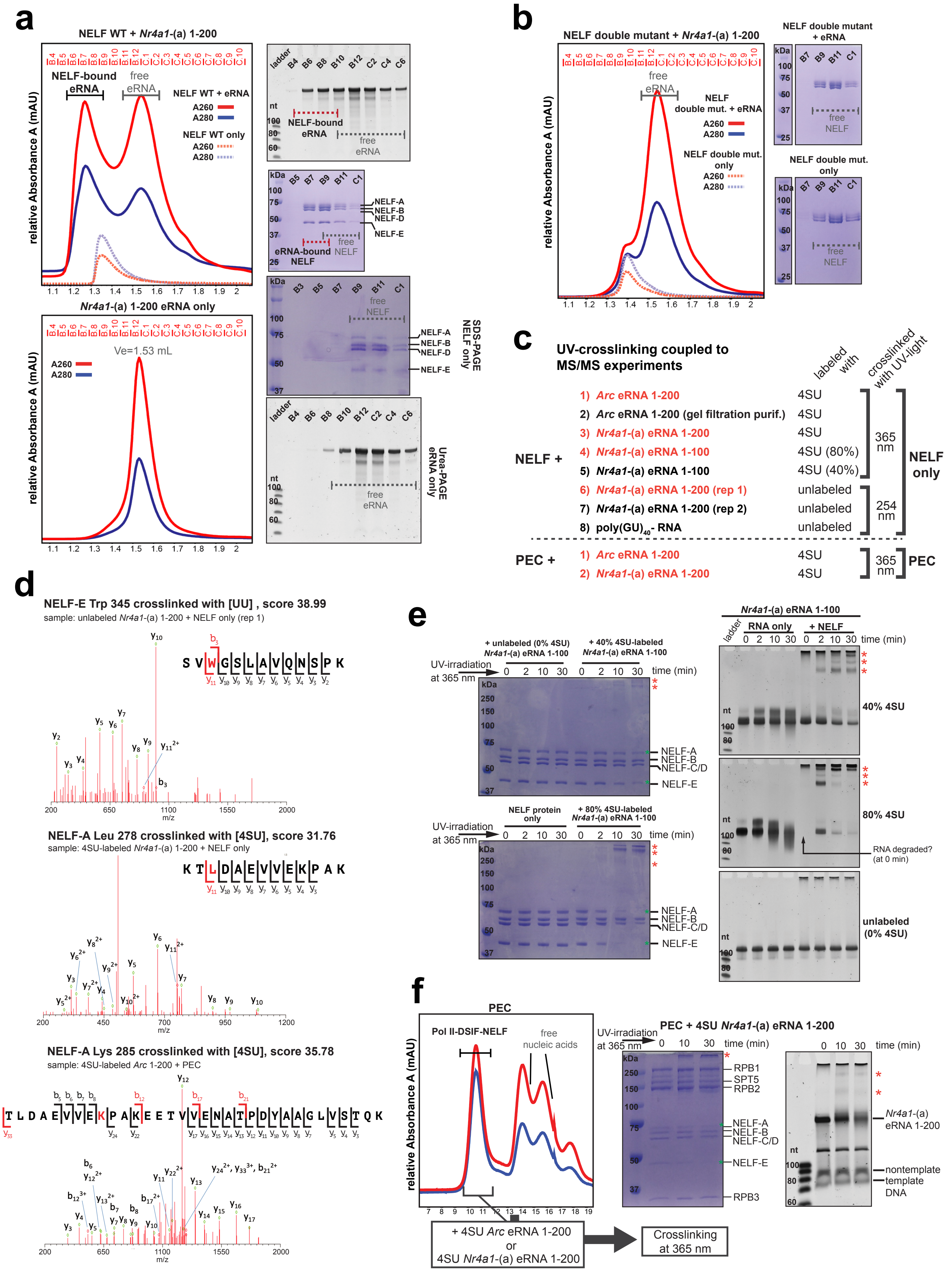

**Supplementary Fig. 4 | UV-crosslinking of eRNAs to the NELF complex and to NELF as part of the PEC.** **a,** Verification of NELF binding to unlabeled *Nr4a1*-(a) eRNA (1-200) by analytical gel filtration (Superose 6 3.2/300 column). The left panel shows the chromatograms of the eRNA + NELF wildtype (WT, solid lines) and NELF WT only (dashed lines) gel filtration (top chromatogram), and an eRNA-only gel filtration (bottom). Chromatograms display the absorbance A in mAU at 280 nm (blue) and 260 nm (red). Each eRNA was pre-incubated with NELF in am 1:1 ratio (300 pmol in 40 µL) for 20 min at 25°C before 25 µL of this reaction was loaded on the column. The right panel shows the corresponding urea PAGE and/or SDS PAGE gels of the respective fractions from each run. The first peak in the eRNA+NELF chromatogram refers to the formed RNA-protein complex, which was confirmed by the analytical gels. These demonstrate a shift of the eRNA and NELF band to an earlier elution volume in comparison to the RNA and protein only gel filtration runs. The samples of the NELF-eRNA complex for the urea PAGE gel were treated with Proteinase K before they were loaded on the gel. Otherwise, we found NELF-eRNA complexes to not run properly into the gel. **b,** The left panel shows the gel filtration chromatogram of *Nr4a1*-(a) eRNA (1-200) preincubated in a 1:1 ratio with the NELF double mutant (solid lines) together with a profile of the protein only run (dashed lines). The elution profile clearly demonstrates that no complex between the eRNA and the NELF double mutant was formed. The right panel shows the corresponding SDS PAGE of the respective fractions of each run. **c,** List of all UV-crosslinked protein-RNA complex samples that were analyzed by mass spectrometry. The crosslinked RNAs were either unlabeled and crosslinked using 254 nm UV-C light or labeled with 4-thiouridine (4SU) and crosslinked at 365 nm UV-A light. In total ten samples were analyzed. For eight samples RNA was crosslinked to the NELF complex ("NELF only" samples), whereas for two samples RNA was crosslinked to the paused elongation complex ("PEC" samples). All crosslinking results can be found in Supplementary Data 4 and 6 (see Supplementary Data 5,7 for data summaries). The crosslinking results of the samples highlighted in red are presented in Fig. 4. Additional note: The unlabeled *Nr4a1*-(a) 1-200 eRNA + NELF experiment was carried out in two replicates (rep 1 and rep 2), whereas in case of the 4SU-labeled *Arc* 1-200 + NELF only experiment only for one of the two crosslinked samples the NELF-eRNA complex was isolated by gel filtration before having been crosslinked (see Methods for a detailed description). **d,** Representative MS/MS fragment spectra of three nucleotide-NELF crosslinks from three different samples that represent three different sorts of protein-RNA crosslinks. Signals corresponding to peptide y- and b- ions are assigned and are marked along the peptide sequence. The signals of common ions, showing no additional mass of a crosslinked nucleotide are marked with a green diamond

(shown black in the peptide sequence), those signals that carry an additional mass of a crosslinked nucleotide are marked with a red diamond (shown red in the peptide sequence). The crosslinked amino acid residue is colored in red. **e,** 4SU labeled *Nr4a1*-(a) eRNA (1-100) efficiently crosslinks to NELF upon irradiation with 365 nm UV light. eRNA + NELF samples were incubated in a 1:1 ratio for 20 min at 25°C before irradiation. Analytical SDS-PAGE (left panel) and urea-PAGE (right panel) show the crosslinking progress for 4SU labeled *Nr4a1*-(a), in which either 40% or 80% of uridines were substituted with 4SU. Protein only and unlabeled (0% 4SU) eRNA only samples serve as controls. Samples resolved on the gels were taken after 0 min (before irradiation), 2, 10 and 30 min of irradiation. **f,** Purification of the PEC and subsequent crosslinking with 4SU labeled *Nr4a1*-(a) eRNA (1-200). The PEC was assembled as described in Methods on the same transcription bubble as used for the transcriptional pause release assays. To remove excess nucleic acids, it was purified by gel filtration (left panel, chromatogram from analytical Superose 6 3.2/300 column). The purified PEC was mixed in a 1:1 ratio with 4SU labeled eRNA and irradiated with 365 nm UV-A light. Samples were taken after 0 (before irradiation), 10 and 30 min of irradiation. The corresponding analytical SDS-PAGE and urea-PAGEs of these samples are shown. Bands of crosslinking products in (e-f) are marked with a red asterisk and protein bands which disappear upon crosslinking are marked with a green asterisk. All urea PAGE gels shown in (a,e,f) are stained with SYBR Gold and all SDS-PAGE gels are stained with Coomassie brilliant blue. Source data for (c) are provided as a Source Data file.

# Supplementary Fig. 5

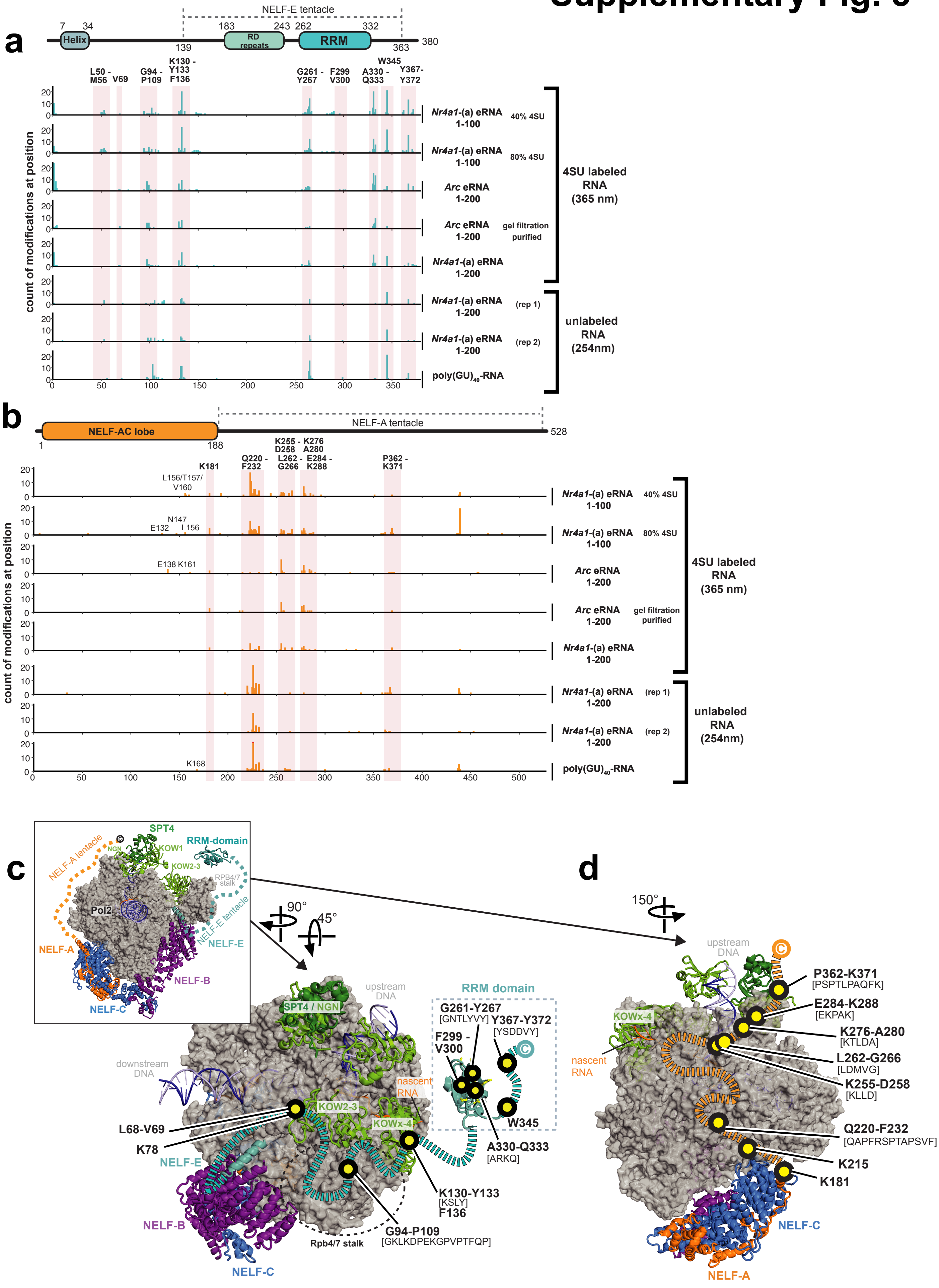

**Supplementary Fig. 5 | RNA predominantly crosslinks to flexible parts of NELF-E and NELF-A. a,** RNA-protein crosslinks plotted along the NELF-E sequence (amino acids 1-380) at each amino acid (aa) position. Plots are displayed for all eight NELF only samples that are listed in Supplementary Fig. 4c. Four plots that are part of Fig. 4b are replicated here to highlight the reproducibility of the crosslink positions amongst all samples. **b,** Same as in **(a)** only for NELF-A. **c,** RNA crosslinks to the structurally unresolved parts of NELF-E (aa 35-261 and aa 333-380) and the NELF-E RRM domain (aa 252-332; PDB code 2JX2) are shown in context of the entire PEC (PDB code 6GML). The position of the unstructured part along the Pol II-DSIF surface is indicated as a dashed line and is based on previous crosslinking data[3]. The encircled C at the end of the dashed line depicts the C-terminus. Crosslinked residues on NELF-E are highlighted as yellow circles and are listed next to the structure. **d,** Analogous to **(c)** only for RNA crosslinks to the structurally unresolved part of NELF-A (NELF-A tentacle; aa 189-528). The views of the PEC displayed in **(c,d)** were generated relative to the canonical front view of the PEC[3], which is shown on top.

# Supplementary Fig. 6

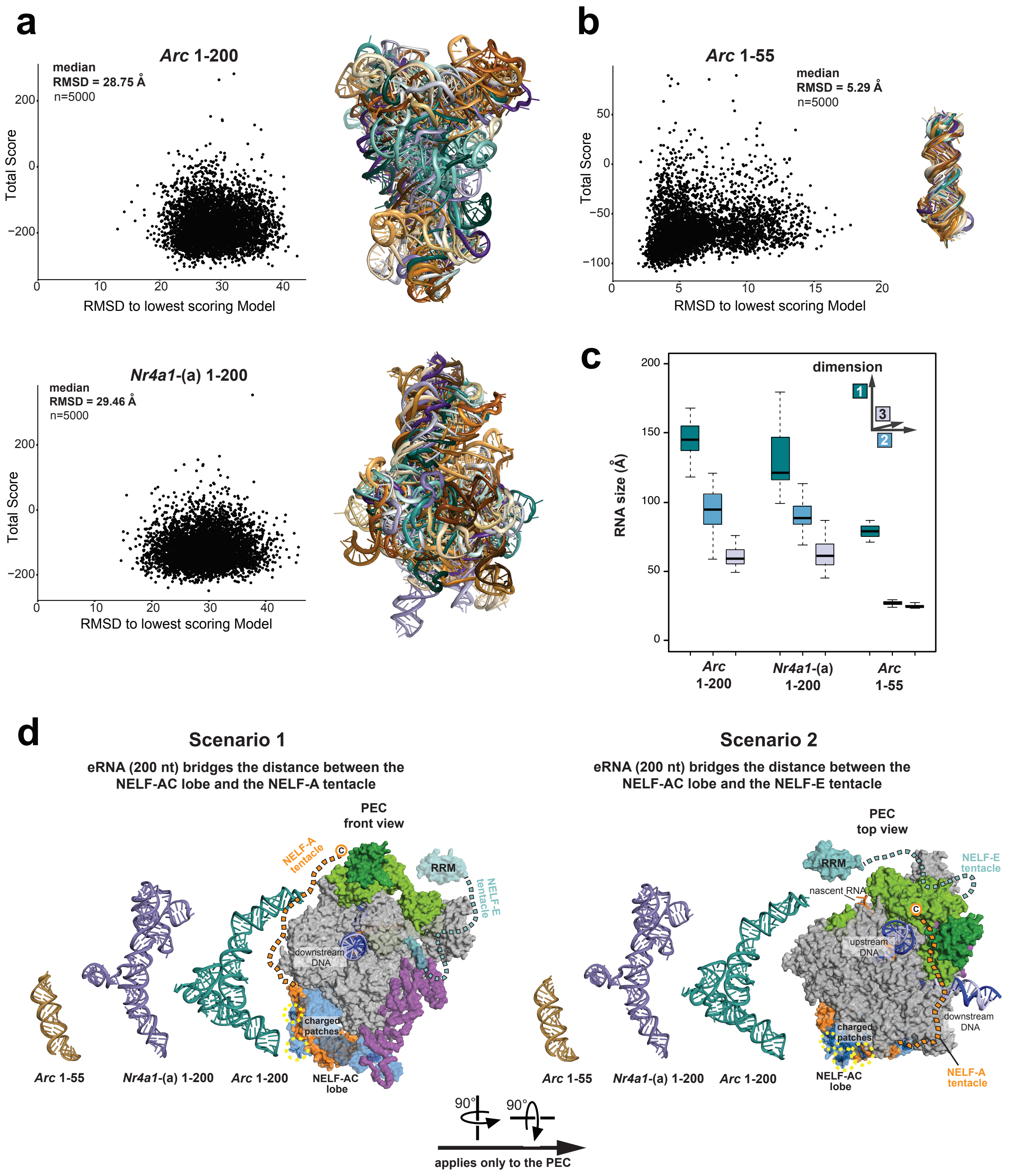

**Supplementary Fig. 6 | 3D structure modeling of 200 nt long eRNAs reveals that their dimensions match the dimensions of the PEC. a,** 3D structure modeling of *Arc* and *Nr4a1*-(a) 1-200 eRNA fragments. The panels on the left show the score-rmsd plots as calculated relatively to the best scoring structure model (described in Methods). Panels on the right show the 20 best scoring structures. In total 5,000 *ab initio* structure models were computed based on the SHAPE-MaP determined secondary structure using Rosetta's *FARFAR2*[4]. **b**, shows the same as in **(a)** only for the *Arc* 1-55 fragment. **c,** Dimensions of the 20 best scoring 3D structures presented in **(a-b)** in (see Methods for description). **d,** Best scoring structure of *Arc* 1-55, *Arc* and *Nr4a1*-(a) 1-200 eRNA fragments is shown next to the PEC (PDB code 6GML) to emphasize that the dimensions of a 200 nucleotide eRNA matches the dimension of the PEC, likely enabling an eRNA to simultaneously bind the NELF-AC lobe and the flexible NELF-A tentacle (Scenario 1, left panel) or the NELF-E tentacle (Scenario 2, right panel). The flexible, unresolved parts of NELF-A and NELF-E are depicted as dashed lines. Source data for (a-c) are provided as a Source Data file.

# Supplementary Fig. 7

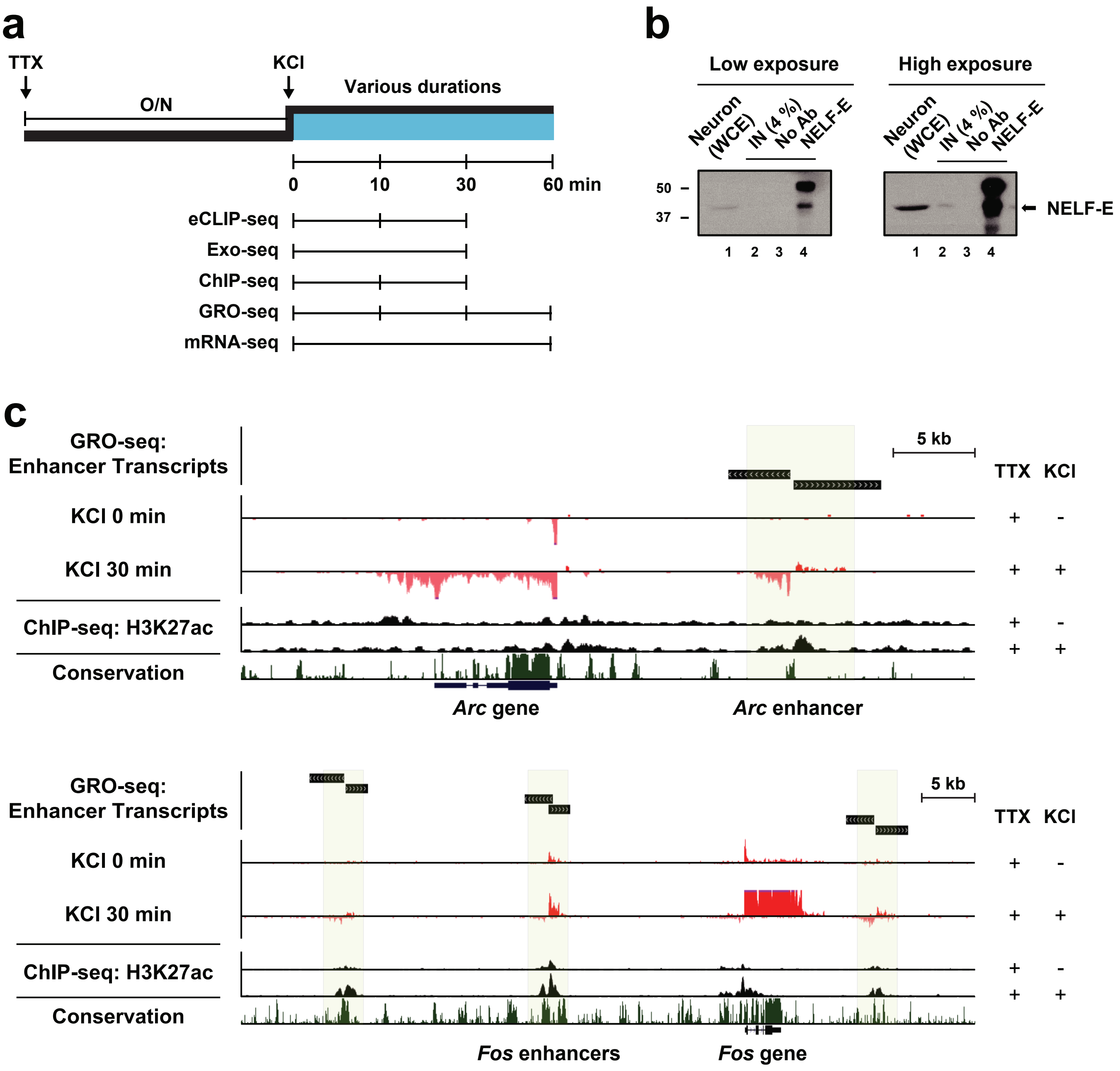

**Supplementary Fig. 7 | Integrative analyses for determining the genome-wide interaction map between NELF and RNA. a**, Experimental scheme for eCLIP-seq, Exo-seq, ChIP-seq, GRO-seq and mRNA-seq *in vivo*. **b**, Western in two different exposures showing the amount of NELF-E pulled down under eCLIP condition (*n* = 1). Source data are provided as a Source Data file. **c**, Example tracks of *de novo* transcript calling on enhancers from GRO-seq that was identified based on the H3K27ac enriched peaks at the activity-induced genes, *Arc* and *Fos* gene loci.

# Supplementary Fig. 8

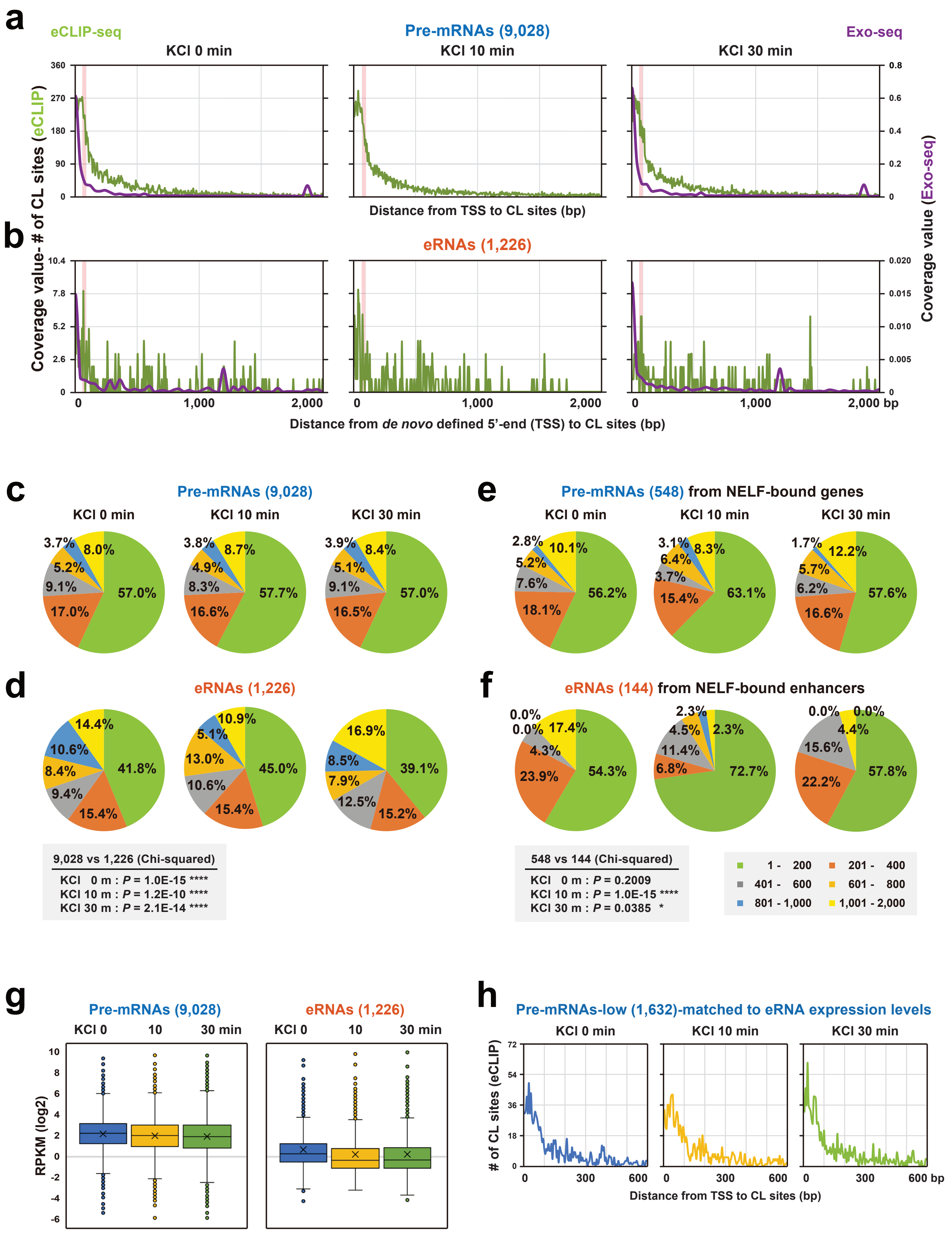

**Supplementary Fig. 8 | NELF interacts with the 5'-end regions of neuronal pre-mRNAs and eRNAs. a** and **b**, The coverage profiles of the crosslinking sites from eCLIP-seq (green line) and Exo-seq reads (purple line) for total annotated pre-mRNAs (9,028) (**a**) and total intergenic eRNAs (1,226) (**b**) – same as Fig. 5b,c except showing a larger downstream region (up to 2 kb) and different time points after KCl stimulation (0, 10 and 30 min) (related to Fig. 5b,c). Note that KCl 10 min was not analyzed in Exo-seq. NELF peak (~60-70 bp downstream of the TSSs) portions (thin red box) from ChIP-seq data are also shown. A total of 7,242 pre-mRNAs having one or more crosslinking sites (4,974, 4,904 and 4,892 from KCl 0, 10 and 30 min) among 9,028 pre-mRNAs were used for these analyzes. The 607 overlapping eRNAs out of 1,226 eRNAs were selected after transcript calling with 30 min KCl GRO-seq sample, and a total of 240 eRNAs having one or more crosslinking sites (124, 127 and 120 from KCl 0, 10 and 30 min) among 607 overlapping eRNAs were used for these analyzes. **c**, **d**, **e** and **f**, Pie chart showing the proportion of the crosslinking sites present in six distance windows (1-200, 201-400, 401-600, 601-800, 801-1,000 or 1,001-2,000 nts) for total annotated (9,028) (**c**) or KCl-up/ NELF-bound (548) (**e**) pre-mRNAs, and total intergenic (1,226) (**d**) or KCl-up/ NELF-bound (144) (**f**) eRNAs at different time points after KCl stimulation (related to Fig. 5f-i). *P*-values were determined by Chi-squared. **g**, The box plot showing RPKM changes (log2) of total pre-mRNAs (9,028) and eRNAs (1,226) at different time points after KCl stimulation (0, 10 and 30 min). Center lines show the medians; box limits indicate the 25th and 75th percentiles; whiskers extend 1.5 times the interquartile range from the 25th and 75th percentiles; X indicates sample means; outliers are represented by dots. **h**, The coverage profiles of the crosslinking sites from eCLIP-seq for selected 1,632 pre-mRNAs. The eRNA expression ranges to be used were determined with values belonging to the 25th and 75th percentiles containing the median based on KCl 10 min in the box plots (**g**), and 1,632 pre-mRNAs with expression levels matching these values were selected from 9,028. Source data for (a-h) are provided as a Source Data file.

# Supplementary Fig. 9

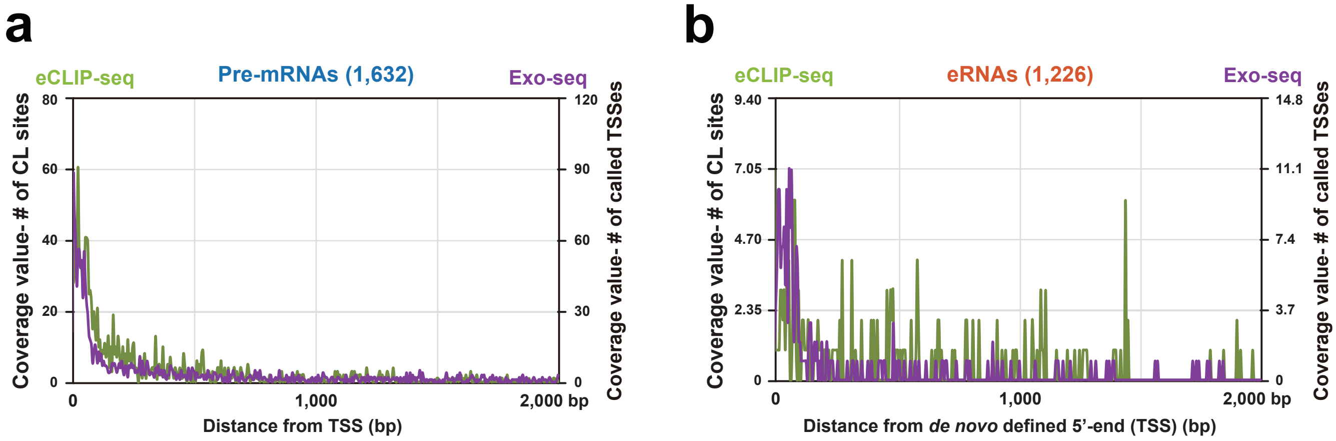

**Supplementary Fig. 9 | eRNA crosslinking results from NELF interactions at various positions along the length of transcribed eRNAs. a** and **b**, Coverage profiles of the crosslinking sites from eCLIP-seq (green line) and the called TSSs (purple line) defined by an Exo-seq read clustering algorithm displaying a region up to 2,000 bp from the TSS (related to Fig. 5b-e). The TSS positions as defined by ENCODE annotation of 708 pre-mRNAs having one or more crosslinking sites out of 1,632 pre-mRNAs whose expression levels were comparable to eRNAs (See also the figure legend of Supplementary Fig. 8h) (**a**) and the *de novo* defined 5'-ends (TSSs) of 120 eRNAs having one or more crosslinking sites out of 1,226 eRNAs determined by *de novo* transcript calling of 30 min KCl GRO-seq sample (**b**) were used for this analysis. Source data for (a and b) are provided as a Source Data file.

# Supplementary Fig. 10

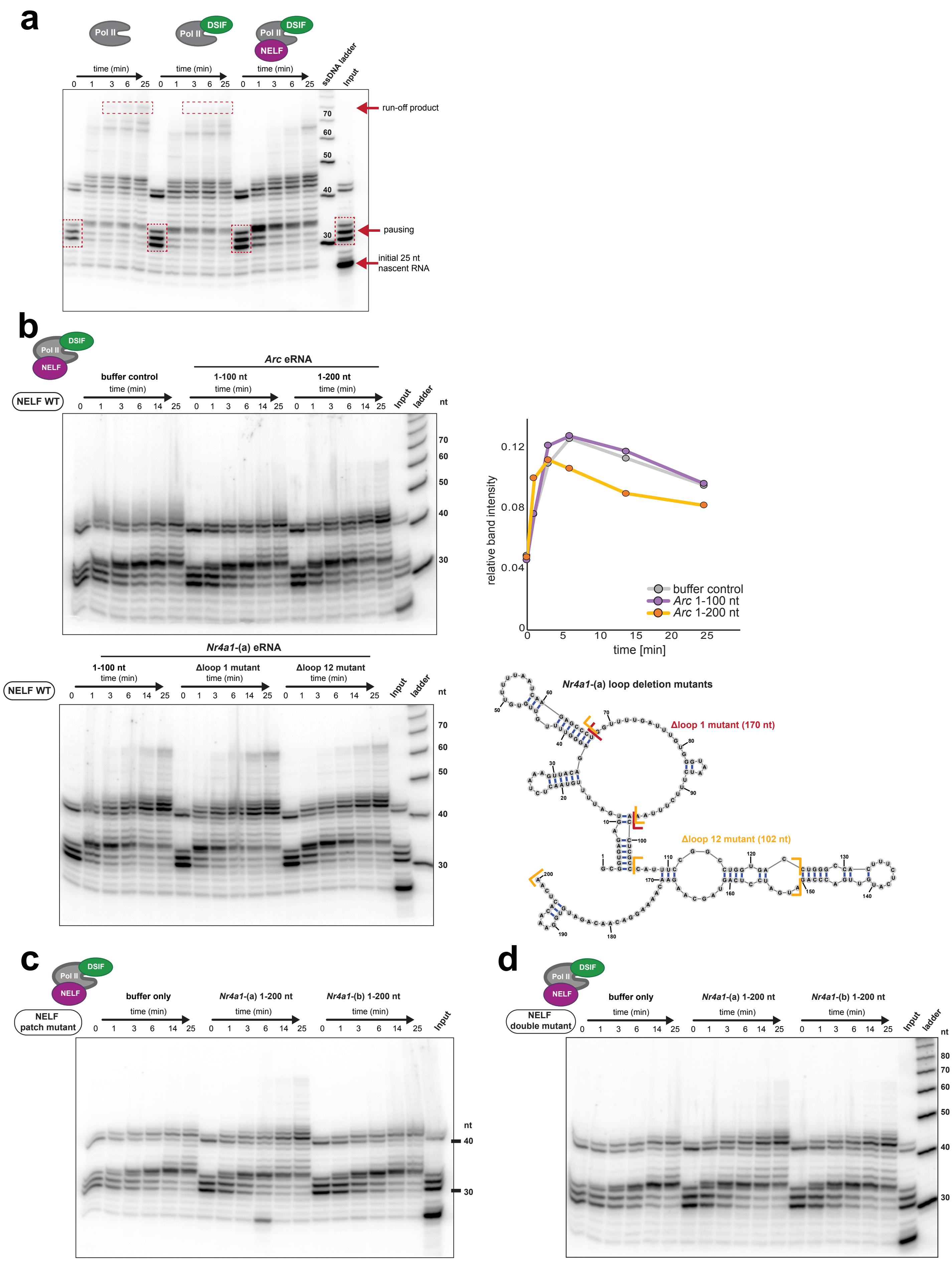

**Supplementary Fig. 10 | DSIF and NELF-dependent pause stabilization and supplementary pause release assays.** All assays were performed as described for Fig. 6a,b. **a,** Transcription assay that verifies pause stabilization in presence of both DSIF and NELF. **b,** Pause release assay with WT NELF and *Arc* (1-100) and (1-200) (top gel) or *Nr4a1*-(a) mutants (bottom gel) (related to Fig. 6b). Next to the top gel the corresponding quantification of the pause release is shown. The quantification for the bottom gel part of Fig. 6b. The secondary structure of *Nr4a1*-(a) (1-200) is shown to the right of the top gel and the deleted regions in the *Nr4a1*-(a) Δloop 1 (red) or the Δloop 12 mutant (orange) are marked in the structure. **c,** Pause release assay with the NELF patch mutant (related to Fig. 6c). **d,** Pause release assay using the NELF double mutant (related to Fig. 6d). Source data for (a-d) are provided as a Source Data file.

# Supplementary Fig. 11

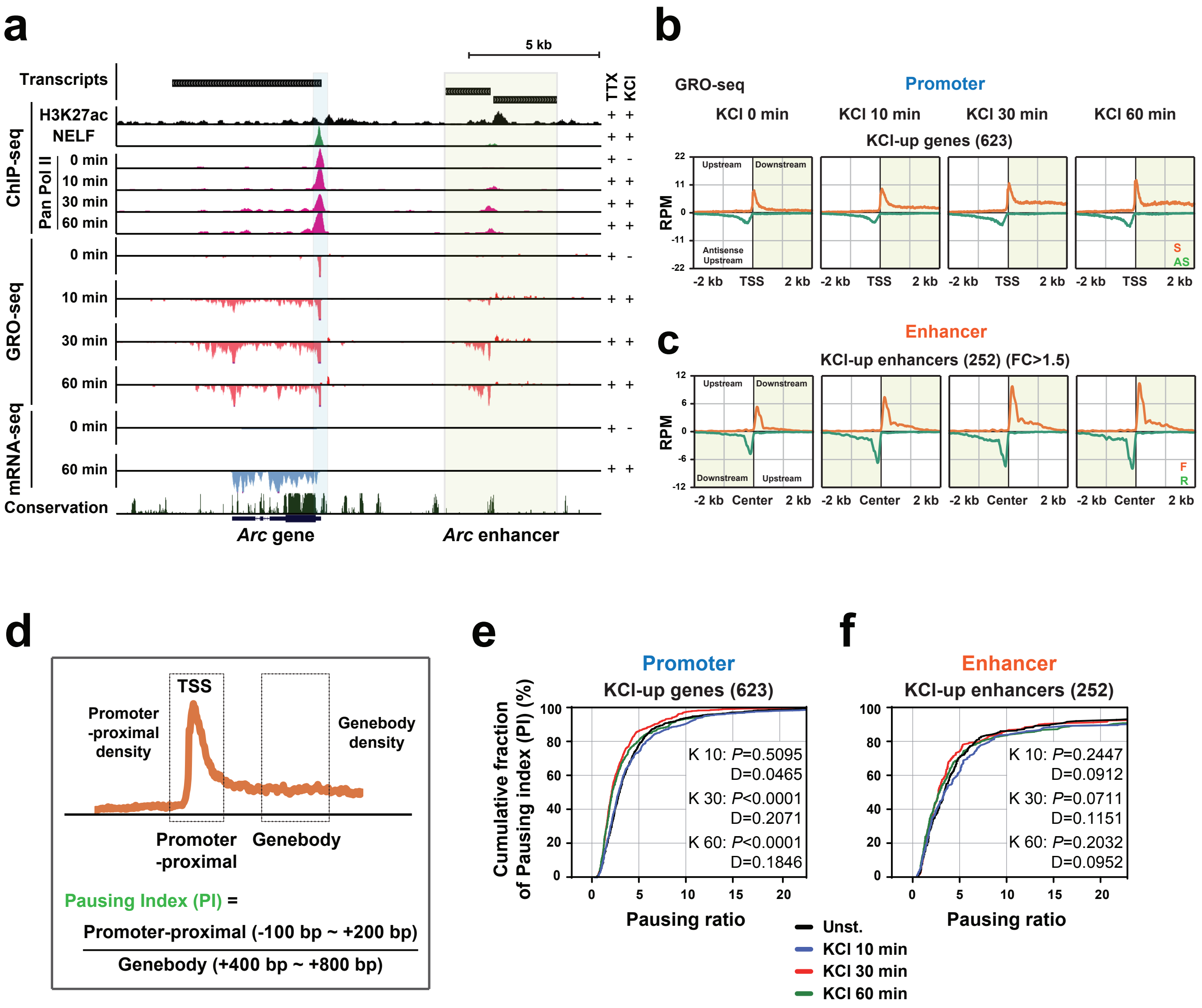

**Supplementary Fig. 11 | NELF-dependent promoter-proximal pausing at neuronal activity-induced genes strongly correlates with the induction levels of these genes *in vivo*.** **a**, Further example of activity induced gene, *Arc* and nearby enhancer (shaded area) (related to Fig. 7a). **b** and **c**, The average GRO-seq profiles at 623 KCl-up genes (**b**) and 252 KCl-up enhancers (FC>1.5) (**c**) (related to Fig. 7b,c). The profiles are described at ±2 kb region centered on the TSSs or the enhancer centers upon 0, 10, 30 and 60 min KCl stimulations. Orange line denotes sense strand (S) at promoters and forward strand (F) at enhancers. Green line denotes anti-sense (AS) at promoters and reverse (R) at enhancers. **d**, Illustration explaining the calculation of the Pausing index (PI) by GRO-seq transcripts at promoter-proximal vs gene body locations. The promoter-proximal region is defined from 100 bp upstream to 200 bp downstream of the TSS. The gene body location is defined from 400 to 800 bp downstream of the TSS (related to Fig. 7e,f). (**e** and **f**), PI profiles of 623 KCl-up genes (**e**) and 252 KCl-up enhancers (**f**). This gene group is analyzed for 4 time points (0, 10, 30 and 60 min KCl). Statistical significance between cumulative probability graphs was determined by the Kolmogorov-Smirnov test. Source data for (b,c,e and f) are provided as a Source Data file.

**SUPPLEMENTARY FIGURE REFERENCES**

1. Siegfried, N. A., Busan, S., Rice, G. M., Nelson, J. A. E. & Weeks, K. M. RNA motif discovery by SHAPE and mutational profiling (SHAPE-MaP). *Nat Methods* 11, 959–965 (2014).

2. Reuter, J. S. & Mathews, D. H. RNAstructure: software for RNA secondary structure prediction and analysis. *BMC Bioinformatics* 11, 129 (2010).

3. Vos, S. M., Farnung, L., Urlaub, H. & Cramer, P. Structure of paused transcription complex Pol II-DSIF-NELF. *Nature* 560, 601–606 (2018).

4. Watkins, A. M., Rangan, R. & Das, R. FARFAR2: Improved De Novo Rosetta Prediction of Complex Global RNA Folds. *Structure* 28, 963-976.e6 (2020).
