# Supplementary material for: Enhancer RNAs stimulate Pol II pause release by harnessing multivalent interactions to NELF
Source: Nat Commun. 2022 May 4;13:2429. doi: 10.1038/s41467-022-29934-w (PMC9068813; doi:10.1038/s41467-022-29934-w)
Supplement: Supplementary file 3 — Description of Additional Supplementary Files [file 41467_2022_29934_MOESM3_ESM.pdf]

Description of Additional Supplementary Files

File Name: Supplementary Data 1
Description: **List of 1,226 *de novo* enhancer transcripts from GRO-seq enhancers.** The super-enhancers (SEs) and typical-enhancers (TEs) were used as the pool of total enhancers. To define *de novo* enhancer transcript calling on enhancers from total GRO-seq, the H3K27ac enriched peaks within ±2 kb regions in the TSS and gene body regions were removed from total enhancers. Then, GRO-seq transcripts which were overlapped with total enhancers were defined as *de novo* enhancer transcripts.

File Name: Supplementary Data 2
Description: **Compiled set of 39 eRNA TSSs.** The table contains the ID/name of the cloned eRNA and the corresponding GRO-seq ID from Supplementary Data 1. It contains further the information about the genomic location of the cloned TSS (chromosome [chr], strand [+/-], start coordinate [start]) and the peak calling ID from TSS call from the two Exo-seq replicates [eTSS_ID_Rep1 and Rep2], originating from KCl-stimulated neuronal samples. Raw sequencing data are available from the GEO superseries GSE163113 (KCl_Rep1 = GSM4972185; KCl_Rep2 = GSM4972186)

File Name: Supplementary Data 3
Description: **Summary of the SHAPE-MaP sequencing statistics for each of the 39 eRNAs.** Median and n-percentile of the read depth and the mutation rates (in percent) are listed for each eRNA and each experimental condition (DMSO- control; 1M7-modified). Data for each eRNA was calculated from the associated *ShapeMapper2* output "_profile.txt" file containing the information for read depth and mutation rates per nucleotide position. Different eRNAs were sequenced in two independent sequencing runs, as stated in the last column of the file (raw sequencing data are available from the GEO superseries with the accession number GSE163113 (Run1 = GSM4972187/GSM4972188; Run2= GSM4972189/GSM4972190)

File Name: Supplementary Data 4
Description: **Protein-RNA Crosslinking mass spectrometry data for 'NELF only' samples.**
Crosslinking data for the eight 'NELF only' samples, that are listed in the supplementary Fig. 4c. Data for each sample is listed in separate excel sheets (sheets are named accordingly to supplementary Fig. 4c). The lists are sorted by descending score of the hit (score cutoff is >20; see Methods).
Description of the relevant columns: 'Id' = Crosslinked peptide sequence – crosslinked residues from the peptide – additional mass shift
'mass shift' = mass shift relative to a not crosslinked peptide, corresponding to the crosslinked mono-/di-/tri-nucleotide'
'Protein1' = crosslinked protein Uniprot ID
'AbsPos1' = absolute position of the crosslinked amino acid residue within the protein
'nseen' = number of spectrums seen for the same crosslink but with different scores (redundancy of crosslinks). Only the spectrum with the highest score is reported.
'score' = score of the hit
The last sheet within the Supplementary Data 4 excel file 'CLIR_MS_mass_shifts_for_R' contains a list of the expected mass shifts for all considered mono-, di- and tri-nucleotide combinations for experiments with unlabeled and 4SU labeled eRNAs and (GU)40 RNA.

File Name: Supplementary Data 5
Description: **Summary of crosslink data from the 'NELF only' samples.** Reports the number of crosslinks (aggregated number of nseen) for each individual protein, referring to target hits and decoy hits from Supplementary Data 4.

File Name: Supplementary Data 6
Description: **Protein-RNA Crosslinking mass spectrometry results for 'PEC' samples.** Crosslinking data for the two 'PEC' samples listed in the supplementary Fig. 4c. Data are listed as described before (see Supplementary Data 4). In contrast to 'NELF only' samples in Supplementary Data 4, here two crosslinking lists correspond to one PEC sample, as samples were measured with two different collision energies (CE23 and CE28).
See Methods section for details.

File Name: Supplementary Data 7
Description: **Summary of crosslink data from the 'PEC' samples.** Reports the number of crosslinks (aggregated number of nseen) for each individual protein, referring to target hits and decoy hits from Supplementary Data 6.
