# Supplementary material for: Enhancer RNAs stimulate Pol II pause release by harnessing multivalent interactions to NELF
Source: Nat Commun. 2022 May 4;13:2429. doi: 10.1038/s41467-022-29934-w (PMC9068813; doi:10.1038/s41467-022-29934-w)
Supplement: Supplementary file 10 — Supplementary Data 7 [file 41467_2022_29934_MOESM10_ESM.zip › Supplementary Data 7.docx]

| **­** |  | **Protein ID** | **Total number of spectra seen (nseen)** |
| --- | --- | --- | --- |
| PEC + *Arc* 1-200 4-sU | NELF | sp\|Q9H3P2\|NELFA_HUMAN | 41 |
|  |  | sp\|Q8WX92\|NELFB_HUMAN | 7 |
|  |  | sp\|Q8IXH7\|NELFD_HUMAN | 3 |
|  |  | sp\|P18615\|NELFE_HUMAN | 90 |
|  | DSIF | sp\|P63272\|SPT4H_HUMAN | 8 |
|  |  | sp\|O00267\|SPT5H_HUMAN | 48 |
|  | Pol II | sp\|P24928\|RPB1_HUMAN | 94 |
|  |  | sp\|P30876\|RPB2_HUMAN | 43 |
|  |  | sp\|P19387\|RPB3_HUMAN | 6 |
|  |  | sp\|O15514\|RPB4_HUMAN | 1 |
|  |  | sp\|P62487\|RPB7_HUMAN | 6 |
|  |  | sp\|P52435\|RPB11_HUMAN | 4 |
|  | contaminants | sp\|P13645\|K1C10_HUMAN | 1 |
|  |  | sp\|Q9U639\|HSP7D_MANSE | 1 |
|  | decoy hits | reverse_sp\|P63272\|SPT4H_HUMAN | 3 |
|  |  | reverse_sp\|O00267\|SPT5H_HUMAN | 2 |
|  |  | reverse_sp\|P30876\|RPB2_HUMAN | 3 |
|  |  | reverse_sp\|P52435\|RPB11_HUMAN | 7 |
| PEC + *Nr4a1*-(a) 1-200 4-sU | NELF | sp\|Q9H3P2\|NELFA_HUMAN | 16 |
|  |  | sp\|Q8WX92\|NELFB_HUMAN | 8 |
|  |  | sp\|Q8IXH7\|NELFD_HUMAN | 2 |
|  |  | sp\|P18615\|NELFE_HUMAN | 56 |
|  | DSIF | sp\|P63272\|SPT4H_HUMAN | 2 |
|  |  | sp\|O00267\|SPT5H_HUMAN | 24 |
|  | Pol II | sp\|P24928\|RPB1_HUMAN | 26 |
|  |  | sp\|P30876\|RPB2_HUMAN | 11 |
|  |  | sp\|P19387\|RPB3_HUMAN | 1 |
|  |  | sp\|P62487\|RPB7_HUMAN | 1 |
|  | contaminants | sp\|Q9U639\|HSP7D_MANSE | 2 |
|  | decoy hits | reverse_sp\|Q9H3P2\|NELFA_HUMAN | 1 |
|  |  | reverse_sp\|Q8WX92\|NELFB_HUMAN | 1 |
|  |  | reverse_sp\|Q8IXH7\|NELFD_HUMAN | 1 |
|  |  | reverse_sp\|P18615\|NELFE_HUMAN | 2 |
|  |  | reverse_sp\|P63272\|SPT4H_HUMAN | 2 |
|  |  | reverse_sp\|O00267\|SPT5H_HUMAN | 2 |
|  |  | reverse_sp\|P24928\|RPB1_HUMAN | 1 |
|  |  | reverse_sp\|P30876\|RPB2_HUMAN | 4 |
|  |  | reverse_sp\|P52435\|RPB11_HUMAN | 7 |
|  |  | reverse_sp\|Q9U639\|HSP7D_MANSE | 2 |
|  |  | reverse_sp\|P13645\|K1C10_HUMAN | 1 |

**Supplementary Table 7**
