# Supplementary material for: Enhancer RNAs stimulate Pol II pause release by harnessing multivalent interactions to NELF
Source: Nat Commun. 2022 May 4;13:2429. doi: 10.1038/s41467-022-29934-w (PMC9068813; doi:10.1038/s41467-022-29934-w)
Supplement: Supplementary file 11 — Reporting Summary [file 41467_2022_29934_MOESM11_ESM.pdf]

nature research

Corresponding author(s): T.-K. Kim: tkkim@postech.ac.kr
C.-D. Kuhn: claus.kuhn@uni-bayreuth.de

Last updated by author(s): Mar 10, 2022

Reporting Summary

Nature Research wishes to improve the reproducibility of the work that we publish. This form provides structure for consistency and transparency in reporting. For further information on Nature Research policies, see our Editorial Policies and the Editorial Policy Checklist.

Statistics

For all statistical analyses, confirm that the following items are present in the figure legend, table legend, main text, or Methods section.

n/a	Confirmed	
☐	☒	The exact sample size (*n*) for each experimental group/condition, given as a discrete number and unit of measurement
☐	☒	A statement on whether measurements were taken from distinct samples or whether the same sample was measured repeatedly
☐	☒	The statistical test(s) used AND whether they are one- or two-sided<br>*Only common tests should be described solely by name; describe more complex techniques in the Methods section.*
☒	☐	A description of all covariates tested
☐	☒	A description of any assumptions or corrections, such as tests of normality and adjustment for multiple comparisons
☐	☒	A full description of the statistical parameters including central tendency (e.g. means) or other basic estimates (e.g. regression coefficient) AND variation (e.g. standard deviation) or associated estimates of uncertainty (e.g. confidence intervals)
☐	☒	For null hypothesis testing, the test statistic (e.g. *F*, *t*, *r*) with confidence intervals, effect sizes, degrees of freedom and *P* value noted<br>*Give P values as exact values whenever suitable.*
☒	☐	For Bayesian analysis, information on the choice of priors and Markov chain Monte Carlo settings
☒	☐	For hierarchical and complex designs, identification of the appropriate level for tests and full reporting of outcomes
☒	☐	Estimates of effect sizes (e.g. Cohen's *d*, Pearson's *r*), indicating how they were calculated

Our web collection on statistics for biologists contains articles on many of the points above.

Software and code

Policy information about availability of computer code

Data collection	Deep sequencing (Exo-seq, ChIP-seq, mRNA-seq, GRO-seq , eCLIP-seq) and SHAPE-MaP data were collected using an Illumina HISEQ 2500 or NEXTseq 500 machine.
Data analysis	Exo-seq:<br>Trim_galore(v 0.6.6) (https://github.com/FelixKrueger/TrimGalore)<br>Cutadapt (v 1.15) (Martin, 2011; https://github.com/marcelm/cutadapt)<br>SortMeRNA (v 2.1) (Kopylova et al., 2012; https://github.com/biocore/sortmerna)<br>Bowtie (v 1.2.2) (Langmead et al., 2009; http://bowtie-bio.sourceforge.net/index.shtml)<br>STAR (v 2.7.8a) (Dobin et al., 2013;https://github.com/alexdobin/STAR)<br>BEDTools (v 2.29.1) (Quinlan and Hall, 2010;https://bedtools.readthedocs.io/en/latest/)<br>TSSCall (Henriques et al., 2018; https://github.com/lavenderca/TSScall)<br>HOMER(v 4.11.1) (http://homer.ucsd.edu/homer/index.html)<br>Ngs.plot(v 2.61) (L. Shen et al. 2014;https://github.com/shenlab-sinai/ngsplotdb)<br>R (v 3.6.3) (R Core Team, 2020; https://www.R-project.org/)<br>gffread (v 0.11.8) (Pertea and Pertea, 2020; https://github.com/gpertea/gffread)<br><br>SHAPE-MaP:<br>ShapeMapper 2 (v 2.1.3) (Busan and Weeks, 2018; https://github.com/Weeks-UNC/shapemapper2)<br>RNAstructure (v 6.0.1) (Reuter and Mathews, 2010; https://rna.urmc.rochester.edu/RNAstructure.html)<br>gffread (v 0.11.8) (Pertea and Pertea, 2020; https://github.com/gpertea/gffread)<br><br>ChIP-seq:<br>Trim_galore(v 0.6.6) (https://github.com/FelixKrueger/TrimGalore)<br>Bowtie2 (v 2.4.4) (Langmead and Salzberg et al., 2012; http://bowtie-bio.sourceforge.net/bowtie2/index.shtml)

SAMtools (v 1.13) (Li et al., 2009; http://samtools.sourceforge.net/)
Sambamba (v 0.8.0) (Tarasov et al., 2015; https://lomereiter.github.io/sambamba/)
DeepTools (v3.5.0) (Ramirez et al., 2014; https://deeptools.readthedocs.io/en/develop/)
MACS2 (v2.2.7.1) (Zhang et al., 2008; https://github.com/taoliu/MACS)
HOMER (Benner Lab; http://homer.ucsd.edu/homer/ngs/)
BEDTools (v. 2.29.1) (Quinlan and Hall, 2010;https://bedtools.readthedocs.io/en/latest/)
ROSE (Whyte et al., 2013; https://bitbucket.org/young_computation/rose)
Bamtools (v 2.5.2) (Barnett et al., 2011; https://github.com/pezmaster31/bamtools)

mRNA-seq:
TopHat (v2.0.12) (Kim et al., 2013; http://ccb.jhu.edu/software/tophat)
SAMtools (v 1.13) (Li et al., 2009; http://samtools.sourceforge.net/)
Picard (v 1.14) Broad Institute (http://broadinstitute.github.io/picard)
HOMER (v 4.11.1) (Benner Lab; http://homer.ucsd.edu/homer/ngs/)
DESeq2 (v 1.34) (Love et al., 2014; https://bioconductor.org/packages/release/bioc/html/DESeq2.html)

GRO-seq:
Trim_galore(v 0.6.6) (https://github.com/FelixKrueger/TrimGalore)
STAR (v 2.7.8a) (Dobin et al., 2013;https://github.com/alexdobin/STAR)
SAMtools (v 1.13) (Li et al., 2009; http://samtools.sourceforge.net/)
HOMER (v 4.11.1) (Benner Lab; http://homer.ucsd.edu/homer/ngs/)
BEDTools (v. 2.29.1) (Quinlan and Hall, 2010;https://bedtools.readthedocs.io/en/latest/)

eCLIP-seq:
Trim_galore(v 0.6.6) (https://github.com/FelixKrueger/TrimGalore)
STAR (v 2.7.8a) (Dobin et al., 2013;https://github.com/alexdobin/STAR)
Sambamba (v 0.8.0) (Tarasov et al., 2015; https://lomereiter.github.io/sambamba/)
BEDTools (v. 2.29.1) (Quinlan and Hall, 2010;https://bedtools.readthedocs.io/en/latest/)
htseq-clip (v 2.10.0) (Anders et al., 2015;https://github.com/EMBL-Hentze-group/htseq-clip)
DeepTools (v3.5.0) (Ramirez et al., 2014; https://deeptools.readthedocs.io/en/develop/)

R custom code(eCLIP-seq Cross-linking distance distribution):

```
### eCLIP-seq KCl 30min CL & TSS distance distribution ###
library(dplyr)
library(ggplot2)

CL <- read.table("eCLIP_K25_1_Prom_9028_CL.bed",sep = "\t")
CL_wa <- read.table("eCLIP_K25_1_Prom_9028_CL.wa.bed",sep = "\t")

tss <- read.table("tss_distance/pre-mRNA_9028_tss_distance_new_min5.bed",sep = "\t")
tss_wa <- read.table("tss_distance/pre-mRNA_9028_tss_distance_new_min5.wa.bed",sep = "\t")


### calculate CL distance ###
m <- cbind(CL,CL_wa)
m_p <- m[m[,6] =="+",]
m_m <- m[m[,6] =="-",]
m_p$V13 <- m_p[,2] - m_p[,8]
m_m$V13 <- m_m[,9] - m_m[,3]
m <- rbind(m_p,m_m)
max(m$V13)
t_m <- table(m$V4,m$V13)
df1 <- table(m$V4,m$V13) %>% as.data.frame()


### calculate tss distance ###
f <- cbind(tss,tss_wa)
f_p <- f[f[,6] =="+",]
f_m <- f[f[,6] =="-",]
f_p$V13 <- f_p[,2] - f_p[,8]
f_m$V13 <- f_m[,9] - f_m[,3]
f <- rbind(f_p,f_m)
head(f)
t_f <- table(f$V4,f$V13)
df2 <- table(f$V4,f$V13) %>% as.data.frame()

kk <- f[f$V4 %in% prom9028_erna$gene_id,]
tss_distance <- colSums(table(kk$V4,kk$V13))
tss_distance <- as.table(colSums(kk))
tss_distance
write.table(tss_distance,"5exo_KCl_30min_rep1_pre-mRNA_9028_eRNA_708_v2.txt",sep = "\t",col.names = F,quote = F,row.names = F)
```

```
t_f <- table(kk$V4,kk$V13)
df2 <- table(f$V4,f$V13) %>% as.data.frame()


prom9028_erna <- read.table("GRO_prom_9028_KCl10min_Q1Q3_1632.txt",sep = "\t",header = T)
names(m)[4] <- c("gene_id")





m2 <- merge(prom9028_erna,m,by="gene_id")

names(m) <-
c("Chr_1","Start_1","End_1","gene_id","Score_1","Strand_1","Chr_2","Start_2","End_2","gene_id2","Score_2","Strand_2","distance")

merge_eRNA2 <- na.omit(merge_erna)

t_m <- table(m2$V4,m2$V13)

df1 <- t_m %>% as.data.frame()

### format conversion CL distance ###
colnames(df1) <- c("tx_name","BP","CL_distance")

d <- data.frame(rep(df1$tx_name[!duplicated(df1$tx_name)], each=2101),
        rep(c(0:2100),times = length(df1$tx_name[!duplicated(df1$tx_name)])))

colnames(d) <- c("tx_name","BP")

d2 <- df1$CL_distance[match(paste0(d$tx_name,d$BP), paste0(df1$tx_name,df1$BP))]

d3 <- cbind(d,d2)

d3$d2 <- ifelse(d3$d2 == 0, NA,no = d3$d2)

colnames(d3) <- c("tx_name","BP","CL_distance")

d3$BP_v2 <- d3$BP-100



### format conversion tss distance ###

colnames(df2) <- c("tx_name","BP","tss_distance")



d <- data.frame(rep(df2$tx_name[!duplicated(df2$tx_name)], each=2101),
        rep(c(0:2100),times = length(df2$tx_name[!duplicated(df2$tx_name)])))

colnames(d) <- c("tx_name","BP")

d2 <- df2$tss_distance[match(paste0(d$tx_name,d$BP), paste0(df2$tx_name,df2$BP))]

d3 <- cbind(d,d2)


d3$d2 <- ifelse(d3$d2 == 0, NA,no = d3$d2)

colnames(d3) <- c("tx_name","BP","tss_distance")

d3$BP_v2 <- d3$BP-100


max(d3$tss_distance,na.rm = T)
unique(df1$tx_name)
head(df2)
d3 <- d3[d3$tx_name %in% gene_708,]
unique(d3$tx_name)
q <- d3 %>% ggplot(aes(x=BP, y = tx_name,size=tss_distance)) +scale_size(breaks = c(0,1),range = c(0,3))+ scale_color_gradient2(midpoint = 0,
low = "lightgrey",high = "red") + theme(legend.position="right") +theme(axis.text.y=element_blank(),axis.title.x = element_text(size=50)) +
geom_point(color="red",shape=15) + theme_classic() + coord_cartesian(xlim = c(-100,2000),expand = TRUE) +scale_x_continuous(breaks =
c(-100,0,500,1000,1500,2000)) +theme(axis.text.y = element_blank())
q2 <- df2 %>% ggplot(aes(x=BP, y = tx_name,size=tss_distance)) +scale_size(breaks = c(0,1),range = c(0,3))+ scale_color_gradient2(midpoint =
```

```
0, low = "lightgrey",high = "red") + theme(legend.position="right") +theme(axis.text.y=element_blank(),axis.title.x = element_text(size=50)) +
geom_point(color="red",shape=15) + theme_classic() + coord_cartesian(xlim = c(-100,2000),expand = TRUE)  +theme(axis.text.y =
element_blank())
pdf("5exo_tss_distance_pre-mRNA_9028_eRNA_exp_1632_CL_708_tss_min5_ver_v2_211221.pdf",height = 20,width = 40,pointsize = 0.1)
q
q2
dev.off()

merged_CL_tss <- merge(d3,df2,by = c("tx_name","BP"),all.x = TRUE)

str(merged_CL_tss)


levels(df2$BP)

merged_CL_tss$CL_distance <- ifelse(merged_CL_tss$CL_distance == 0, NA,no = merged_CL_tss$CL_distance)
merged_CL_tss$tss_distance <- ifelse(merged_CL_tss$tss_distance == 0, NA,no = merged_CL_tss$tss_distance)

head(merged_CL_tss)
uniq_merge <- na.omit(merged_CL_tss)
unique(df1$tx_name)
unique(sort(uniq_merge$tx_name))
max(merged_CL_tss$CL_distance,na.rm = T)




p <- merged_CL_tss %>% ggplot(aes(x=BP_v2, y = tx_name,size=CL_distance)) +scale_size(breaks = c(1,2,3,4,5,6),range = c(7,15))+
scale_color_gradient2(midpoint = 1, low = "lightgrey",high = "black") + theme(legend.position="right")
+theme(axis.text.y=element_blank(),axis.title.x = element_text(size=50)) + geom_point() + theme_classic() + coord_cartesian(xlim =
c(-100,2000),expand = TRUE) +scale_x_continuous(breaks = c(-100,0,500,1000,1500,2000)) +theme(axis.text.y = element_blank())
q <- merged_CL_tss %>% ggplot(aes(x=BP_v2, y = tx_name,size=tss_distance)) +scale_size(breaks = c(0,1),range = c(0,7))+
scale_color_gradient2(midpoint = 0, low = "lightgrey",high = "red") + theme(legend.position="right")
+theme(axis.text.y=element_blank(),axis.title.x = element_text(size=50)) + geom_point(color="red",shape=15) + theme_classic() +
coord_cartesian(xlim = c(-100,2000),expand = TRUE) +scale_x_continuous(breaks = c(-100,0,500,1000,1500,2000)) +theme(axis.text.y =
element_blank())
p2 <- merged_CL_tss %>% ggplot(aes(x=BP_v2, y = tx_name,size=CL_distance)) +scale_size(breaks = c(1,2,3,4,5,6),range = c(7,15))+
scale_color_gradient2(midpoint = 1, low = "lightgrey",high = "black") + theme(legend.position="right")
+theme(axis.text.y=element_blank(),axis.title.x = element_text(size=50)) + geom_point() + theme_classic() + coord_cartesian(xlim =
c(-100,600),expand = TRUE) +scale_x_continuous(breaks = c(-100,0,100,600)) +theme(axis.text.y = element_blank())
q2 <- merged_CL_tss %>% ggplot(aes(x=BP_v2, y = tx_name,size=tss_distance)) +scale_size(breaks = c(0,1),range = c(0,7))+
scale_color_gradient2(midpoint = 0, low = "lightgrey",high = "red") + theme(legend.position="right")
+theme(axis.text.y=element_blank(),axis.title.x = element_text(size=50)) + geom_point(color="red",shape=15) + theme_classic() +
coord_cartesian(xlim = c(-100,600),expand = TRUE) +scale_x_continuous(breaks = c(-100,0,100,600)) +theme(axis.text.y = element_blank())


pdf("eCLIP+tss_distance_pre-mRNA_9028_eRNA_exp_1632_CL_708_tss_min5_ver_v2_211220.pdf",height = 20,width = 40,pointsize = 0.1)
p
q
p2
q2
dev.off()






####Custom R code for Fig. 2j #### Do Boxplot for eRNAs 1:1000 & Bins (200nt)
library(seqinr)

BinList <- read.fasta(file = "Selected_eRNA-Clones_TSSsites_Filtered_binned200bp.fa", forceDNAtolower = T, seqtype = "DNA", as.string = T)
eRNA_1kList <- read.fasta(file = "Selected_eRNA-Clones_TSSsites_Filtered_1kb.fa", forceDNAtolower = T, seqtype = "DNA", as.string = T)

#sort Bins by order
BinList <- BinList[c(1:10,15,14,13,12,11,16:30,40,38,36,34,32,39,37,35,33,31,50,48,46,44,42,49,47,45,43,41,51:65,70,69,68,67,66,
            71,72,74,76,78,73,75,77,79,80,81:90,95,94,93,92,91,100,99,98,97,96,105,104,103,102,101,110,109,108,107,106,
            111:115,120,119,118,117,116,121:135,140,139,138,137,136,145,144,143,142,141,146:155,160,159,158,157,156,161:165,
            170,169,168,167,166,175,174,173,172,171)]

Bins <- unlist(BinList, use.names = F)
eRNA1k <- unlist(eRNA_1kList, use.names = F)

library(stringr)
Bins_BaseCounts <- sapply(c("a","g","c","t"), function(nuc) str_count(Bins, fixed(nuc)))
eRNA1k_BaseCounts <- sapply(c("a","g","c","t"), function(nuc) str_count(eRNA1k, fixed(nuc)))
```

```
colnames(Bins_BaseCounts) = c("A","G","C","T")
colnames(eRNA1k_BaseCounts) = c("A","G","C","T")

percentage <- function(x)(x/sum(x)*100)
Bins_BasePct <- t(apply(Bins_BaseCounts,1,percentage))
eRNA1k_BasePct <- t(apply(eRNA1k_BaseCounts,1,percentage))

Bins_BasePct <- as.data.frame(Bins_BasePct)
Bins_BasePct$Name <- names(BinList)
eRNA1k_BasePct <- as.data.frame(eRNA1k_BasePct)
eRNA1k_BasePct$Name <- names(eRNA_1kList)

Bin1_BasePct <- data.frame(A=0,G=0,C=0,T=0,Name=0)
Bin2_BasePct <- data.frame(A=0,G=0,C=0,T=0,Name=0)
Bin3_BasePct <- data.frame(A=0,G=0,C=0,T=0,Name=0)
Bin4_BasePct <- data.frame(A=0,G=0,C=0,T=0,Name=0)
Bin5_BasePct <- data.frame(A=0,G=0,C=0,T=0,Name=0)

for(i in 0:34){
  Bin1_BasePct <- rbind(Bin1_BasePct, Bins_BasePct[1+i*5,])
  Bin2_BasePct <- rbind(Bin2_BasePct, Bins_BasePct[2+i*5,])
  Bin3_BasePct <- rbind(Bin3_BasePct, Bins_BasePct[3+i*5,])
  Bin4_BasePct <- rbind(Bin4_BasePct, Bins_BasePct[4+i*5,])
  Bin5_BasePct <- rbind(Bin5_BasePct, Bins_BasePct[5+i*5,])
}

Bin1_BasePct <- Bin1_BasePct[-1,]
Bin2_BasePct <- Bin2_BasePct[-1,]
Bin3_BasePct <- Bin3_BasePct[-1,]
Bin4_BasePct <- Bin4_BasePct[-1,]
Bin5_BasePct <- Bin5_BasePct[-1,]


eRNA1k_BasePct <- as.data.frame(eRNA1k_BasePct)
eRNA1k_BasePct$Type =c("1-1000")

Bin1_BasePct<- as.data.frame(Bin1_BasePct)
Bin1_BasePct$Type = c("1-200")
Bin2_BasePct<- as.data.frame(Bin2_BasePct)
Bin2_BasePct$Type = c("201-400")
Bin3_BasePct<- as.data.frame(Bin3_BasePct)
Bin3_BasePct$Type = c("401-600")
Bin4_BasePct<- as.data.frame(Bin4_BasePct)
Bin4_BasePct$Type = c("601-800")
Bin5_BasePct<- as.data.frame(Bin5_BasePct)
Bin5_BasePct$Type = c("801-1000")


Percentage_Plot <- rbind(eRNA1k_BasePct, Bin1_BasePct)
Percentage_Plot <- rbind(Percentage_Plot, Bin2_BasePct)
Percentage_Plot <- rbind(Percentage_Plot, Bin3_BasePct)
Percentage_Plot <- rbind(Percentage_Plot, Bin4_BasePct)
Percentage_Plot <- rbind(Percentage_Plot, Bin5_BasePct)

library("reshape2")
PercentagePlot_final <- melt(Percentage_Plot[,c(1:4,6)], "Type")

PercentagePlot_final$Type <- as.factor(PercentagePlot_final$Type)

library(ggplot2)
#pdf(file = "BaseContent_Comparison_CloneBins_TSS-Starts.pdf", width = 10, height = 8)
ggplot(PercentagePlot_final, aes(x = Type, y = value, fill= variable))+
  geom_boxplot(aes(fill=variable),
               outlier.shape = NA, outlier.size = 1)+ ylab("Percent")+
  theme(panel.grid.major = element_blank(), panel.grid.minor = element_blank(), panel.background = element_blank(), axis.line =
element_line(colour = "black"))+
  scale_fill_manual(values = c("coral3","darkgoldenrod3","cadetblue","darkolivegreen3"))+
  guides(fill=guide_legend(title = "Nucleotide"))
#dev.off()

write.table(Percentage_Plot, file = "./BinData_Nucleotides.csv", col.names = T, sep = "\t", row.names = F)

#####Test significance in Bins
##Pairwise t-test
#replace all instances of BinX_BasePct by the bin to be tested
```

```
A_count <- as.data.frame(Bin1_BasePct[,1])
colnames(A_count) = c("Pct")
A_count$Base <- c("A")
G_count <- as.data.frame(Bin1_BasePct[,2])
colnames(G_count) <- c("Pct")
G_count$Base <- c("G")
C_count <- as.data.frame(Bin1_BasePct[,3])
colnames(C_count) <- c("Pct")
C_count$Base <- c("C")
T_count <- as.data.frame(Bin1_BasePct[,4])
colnames(T_count) <- c("Pct")
T_count$Base <- c("T")

Base_CountsBin1 <- rbind(A_count,C_count,G_count,T_count)
Base_CountsBin1$Pct <- Base_CountsBin1$Pct*2.00
colnames(Base_CountsBin1) = c("Count","Base")
pairwise.t.test(Base_CountsBin1$Count, Base_CountsBin1$Base, p.adjust.method = "BH")
```

For manuscripts utilizing custom algorithms or software that are central to the research but not yet described in published literature, software must be made available to editors and reviewers. We strongly encourage code deposition in a community repository (e.g. GitHub). See the Nature Research guidelines for submitting code & software for further information.

Data

Policy information about availability of data

All manuscripts must include a data availability statement. This statement should provide the following information, where applicable:

- Accession codes, unique identifiers, or web links for publicly available datasets
- A list of figures that have associated raw data
- A description of any restrictions on data availability

The Exo-seq, GRO-seq, ChIP- (NELF-A, NELF-E and Pan Pol II)-seq, eCLIP-seq, and SHAPE-MaP data generated in this study have been deposited in the NCBI Gene Expression Omnibus (GEO) database under accession code GSE163113 [https://www.ncbi.nlm.nih.gov/geo/query/acc.cgi?acc=GSE163113]. Crosslinking mass spectrometry data generated in this study have been deposited to the ProteomeXchange Consortium via the PRIDE partner repository (dataset identifier PXD030569) [http://www.ebi.ac.uk/pride/archive/projects/PXD030569]86. The mRNA-seq data used in this study had previously been generated and are available from in the GEO under accession code GSE139309 (GSM4137778-GSM4137789) [https://www.ncbi.nlm.nih.gov/geo/query/acc.cgi?acc=GSE139309]. ChIP-(H3K27ac, Pol II, and CBP)-seq data used in this study are available in GEO under the accession codes GSE60192 [https://www.ncbi.nlm.nih.gov/geo/query/acc.cgi?acc=GSE60192] and GSE21161 [https://www.ncbi.nlm.nih.gov/geo/query/acc.cgi?acc=GSE21161]. Source data are provided with this paper.

Field-specific reporting

Please select the one below that is the best fit for your research. If you are not sure, read the appropriate sections before making your selection.

☒ Life sciences ☐ Behavioural & social sciences ☐ Ecological, evolutionary & environmental sciences

For a reference copy of the document with all sections, see nature.com/documents/nr-reporting-summary-flat.pdf

Life sciences study design

All studies must disclose on these points even when the disclosure is negative.

Sample size	No pre-determine sample-size calculations were performed. The sample size was based on the literature (Kim et al., Nature, 2010; Kim et al., Sci Adv, 2021) that performed the similar experiments. Sample size for 1-2 biological replicates of sequencing data (Exo-seq, SHAPE, GRO-seq, ChIP-seq, mRNA-seq, eCLIP-seq,) in this study were used according to common practice.
Data exclusions	No data were excluded from analysis.
Replication	Experiments were performed 2-3 times. Similar observation was obtained for each replicate. Representative result was shown in the figures.
Randomization	No randomization was performed, because there were not treatment groups. Most of the comparisons were made between deterministic states, time course.
Blinding	ChIP-seq experiments were not blinded to the definition of genome location of histone modifications and transcription-related factors, and expression data from RNA-seq data and interaction data between protein and RNA of eCLIP-seq were not supervised.

Behavioural & social sciences study design

All studies must disclose on these points even when the disclosure is negative.

Study description	*Briefly describe the study type including whether data are quantitative, qualitative, or mixed-methods (e.g. qualitative cross-sectional, quantitative experimental, mixed-methods case study).*
Research sample	*State the research sample (e.g. Harvard university undergraduates, villagers in rural India) and provide relevant demographic information (e.g. age, sex) and indicate whether the sample is representative. Provide a rationale for the study sample chosen. For studies involving existing datasets, please describe the dataset and source.*
Sampling strategy	*Describe the sampling procedure (e.g. random, snowball, stratified, convenience). Describe the statistical methods that were used to predetermine sample size OR if no sample-size calculation was performed, describe how sample sizes were chosen and provide a rationale for why these sample sizes are sufficient. For qualitative data, please indicate whether data saturation was considered, and what criteria were used to decide that no further sampling was needed.*
Data collection	*Provide details about the data collection procedure, including the instruments or devices used to record the data (e.g. pen and paper, computer, eye tracker, video or audio equipment) whether anyone was present besides the participant(s) and the researcher, and whether the researcher was blind to experimental condition and/or the study hypothesis during data collection.*
Timing	*Indicate the start and stop dates of data collection. If there is a gap between collection periods, state the dates for each sample cohort.*
Data exclusions	*If no data were excluded from the analyses, state so OR if data were excluded, provide the exact number of exclusions and the rationale behind them, indicating whether exclusion criteria were pre-established.*
Non-participation	*State how many participants dropped out/declined participation and the reason(s) given OR provide response rate OR state that no participants dropped out/declined participation.*
Randomization	*If participants were not allocated into experimental groups, state so OR describe how participants were allocated to groups, and if allocation was not random, describe how covariates were controlled.*

Ecological, evolutionary & environmental sciences study design

All studies must disclose on these points even when the disclosure is negative.

Study description	*Briefly describe the study. For quantitative data include treatment factors and interactions, design structure (e.g. factorial, nested, hierarchical), nature and number of experimental units and replicates.*
Research sample	*Describe the research sample (e.g. a group of tagged Passer domesticus, all Stenocereus thurberi within Organ Pipe Cactus National Monument), and provide a rationale for the sample choice. When relevant, describe the organism taxa, source, sex, age range and any manipulations. State what population the sample is meant to represent when applicable. For studies involving existing datasets, describe the data and its source.*
Sampling strategy	*Note the sampling procedure. Describe the statistical methods that were used to predetermine sample size OR if no sample-size calculation was performed, describe how sample sizes were chosen and provide a rationale for why these sample sizes are sufficient.*
Data collection	*Describe the data collection procedure, including who recorded the data and how.*
Timing and spatial scale	*Indicate the start and stop dates of data collection, noting the frequency and periodicity of sampling and providing a rationale for these choices. If there is a gap between collection periods, state the dates for each sample cohort. Specify the spatial scale from which the data are taken*
Data exclusions	*If no data were excluded from the analyses, state so OR if data were excluded, describe the exclusions and the rationale behind them, indicating whether exclusion criteria were pre-established.*
Reproducibility	*Describe the measures taken to verify the reproducibility of experimental findings. For each experiment, note whether any attempts to repeat the experiment failed OR state that all attempts to repeat the experiment were successful.*
Randomization	*Describe how samples/organisms/participants were allocated into groups. If allocation was not random, describe how covariates were controlled. If this is not relevant to your study, explain why.*
Blinding	*Describe the extent of blinding used during data acquisition and analysis. If blinding was not possible, describe why OR explain why blinding was not relevant to your study.*

Did the study involve field work? ☐ Yes ☐ No

Field work, collection and transport

Field conditions	*Describe the study conditions for field work, providing relevant parameters (e.g. temperature, rainfall).*
Location	*State the location of the sampling or experiment, providing relevant parameters (e.g. latitude and longitude, elevation, water depth).*
Access & import/export	*Describe the efforts you have made to access habitats and to collect and import/export your samples in a responsible manner and in compliance with local, national and international laws, noting any permits that were obtained (give the name of the issuing authority, the date of issue, and any identifying information).*
Disturbance	*Describe any disturbance caused by the study and how it was minimized.*

Reporting for specific materials, systems and methods

We require information from authors about some types of materials, experimental systems and methods used in many studies. Here, indicate whether each material, system or method listed is relevant to your study. If you are not sure if a list item applies to your research, read the appropriate section before selecting a response.

Materials & experimental systems

n/a	Involved in the study
☐	☒ Antibodies
☒	☐ Eukaryotic cell lines
☒	☐ Palaeontology and archaeology
☐	☒ Animals and other organisms
☒	☐ Human research participants
☒	☐ Clinical data
☒	☐ Dual use research of concern

Methods

n/a	Involved in the study
☐	☒ ChIP-seq
☒	☐ Flow cytometry
☒	☐ MRI-based neuroimaging

Antibodies

Antibodies used

anti-NELF-A (A-20) antibody, Santa Cruz Biotech, sc-23599, mouse monoclonal antibody
anti-NELF-E antibody, Abcam ,ab170104, Rabbit monoclonal [EPR11600] antibody
anti-Pol II (N-20) antibody, Santa Cruz Biotech, sc-899X, rabbit polyclonal antibody
anti-SPT5 (D-3 X) antibody, Santa Cruz Biotech, sc-133217X, Lot: G2518, mouse monoclonal antibody

Validation

anti-NELF-A antibody is a mouse monoclonal IgG2b κ NELF-A antibody raised against amino acids 92-300 mapping near the N-terminus of NELF-A of human origin. Two µg of this antibody per 12 million cells was used in ChIP experiment. This antibody was used to assess the NELF-A level expressed in the different mammalian lines. This antibody was successfully used in numerous peer-reviewed studies (e.g. Wang et al., Cell Rep 34: 108759; Rivas et al., Mol. Cell. Biol. 41: e0017121

anti-NELF-E antibody is purified a Rabbit Monoclonal IgG raised against recombinant fragment corresponding to Human NELFe. Two µg of this antibody per 12 million cells was used in ChIP experiment and 0.5 µg (2 µL of undiluted) were used per sample for Gel Supershift assays). This antibody was successfully used in numerous peer-reviewed studies (e.g. Takahashi et al., Nat Commun, 26;11(1):1063, 2020; Aoi et al., Mol Cell, 16;78(2):261-274.e5,2020

anti-Pol II (N-20) antibody is an affinity purified rabbit polyclonal antibody raised against a peptide mapping at the N-terminus of Pol II of mouse origin. Two µg of this antibody per 12 million cells was used in ChIP experiment. This antibody was used to assess the RPB1 level expressed in the different mammalian lines. This antibody was successfully used in numerous peer-reviewed studies (e.g. Emmett et al., Nature 546, 544-548, 2017; Dieuleveult et al., Nature 530, 113-6, 2016; Forget et al., NAR 41, 6881-6891, 2015.)

anti-NELF-E (see above) and anti-SPT5 (0.72 µg; 2 µL of 1:5.5 dilution per sample) antibodies were used for Gel Supershift assays to detect whether NELF and/or DSIF is bound to Pol II, in order to validate the EMSA results. SPT5 (D-3) is a mouse monoclonal antibody raised against amino acids 61-360 mapping near the N-terminus of SPT5 of human origin. Anti-SPT5 (D-3) X TransCruz (a higher concentrated version) that was used, is recommended for Gel Supershift and ChIP applications by the manufacturer (Santa Cruz Biotech). Furthermore the antibody was successfully used in numerous peer-reviewed studies for ChIP and ChIP-nexus experiments (e.g. Baluapuri, A., et al, Mol. Cell 74: 674-687.e11, 2019; Mylonas, C., et al., Nat. Struct. Mol. Biol. 28:435-442, 2021; Studniarek, C., et al., Cell Rep. 35: 108965, 2021)

Eukaryotic cell lines

Policy information about cell lines

Cell line source(s)	*State the source of each cell line used.*
Authentication	*Describe the authentication procedures for each cell line used OR declare that none of the cell lines used were authenticated.*

Mycoplasma contamination	*Confirm that all cell lines tested negative for mycoplasma contamination OR describe the results of the testing for mycoplasma contamination OR declare that the cell lines were not tested for mycoplasma contamination.*
Commonly misidentified lines (See ICLAC register)	*Name any commonly misidentified cell lines used in the study and provide a rationale for their use.*

Palaeontology and Archaeology

Specimen provenance	*Provide provenance information for specimens and describe permits that were obtained for the work (including the name of the issuing authority, the date of issue, and any identifying information).*
Specimen deposition	*Indicate where the specimens have been deposited to permit free access by other researchers.*
Dating methods	*If new dates are provided, describe how they were obtained (e.g. collection, storage, sample pretreatment and measurement), where they were obtained (i.e. lab name), the calibration program and the protocol for quality assurance OR state that no new dates are provided.*

☐ Tick this box to confirm that the raw and calibrated dates are available in the paper or in Supplementary Information.

Ethics oversight	*Identify the organization(s) that approved or provided guidance on the study protocol, OR state that no ethical approval or guidance was required and explain why not.*

Note that full information on the approval of the study protocol must also be provided in the manuscript.

Animals and other organisms

Policy information about studies involving animals; ARRIVE guidelines recommended for reporting animal research

Laboratory animals	Mice (Mus musculus) were housed in LD12:12 (12 light cycles/12 dark cycles) conditions at 40-60% humidity and a temperature of 65-75°F (18-23°C). All mice were on a C57BL/6J genetic background (male and female). Timed matings were set up with males and females from 6 to 26 weeks of age and E16-E18 embryos of age were used for experiments.
Wild animals	This study did not involve wild animals.
Field-collected samples	This study did not involve field-collected samples.
Ethics oversight	Valid project licenses were obtained and monitored by the Institutional Animal Care and Use Committee (IACUC) committee at Pohang University of Science and Technology (POSTECH) (Pohang, Gyeongbuk, 37673, Republic of Korea).

Note that full information on the approval of the study protocol must also be provided in the manuscript.

Human research participants

Policy information about studies involving human research participants

Population characteristics	*Describe the covariate-relevant population characteristics of the human research participants (e.g. age, gender, genotypic information, past and current diagnosis and treatment categories). If you filled out the behavioural & social sciences study design questions and have nothing to add here, write "See above."*
Recruitment	*Describe how participants were recruited. Outline any potential self-selection bias or other biases that may be present and how these are likely to impact results.*
Ethics oversight	*Identify the organization(s) that approved the study protocol.*

Note that full information on the approval of the study protocol must also be provided in the manuscript.

Clinical data

Policy information about clinical studies

All manuscripts should comply with the ICMJE guidelines for publication of clinical research and a completed CONSORT checklist must be included with all submissions.

Clinical trial registration	*Provide the trial registration number from ClinicalTrials.gov or an equivalent agency.*
Study protocol	*Note where the full trial protocol can be accessed OR if not available, explain why.*
Data collection	*Describe the settings and locales of data collection, noting the time periods of recruitment and data collection.*
Outcomes	*Describe how you pre-defined primary and secondary outcome measures and how you assessed these measures.*

Dual use research of concern

Policy information about dual use research of concern

Hazards

Could the accidental, deliberate or reckless misuse of agents or technologies generated in the work, or the application of information presented in the manuscript, pose a threat to:

No	Yes	
☒	☐	Public health
☒	☐	National security
☒	☐	Crops and/or livestock
☒	☐	Ecosystems
☐	☒	Any other significant area

Hazards — *Please describe the agents/technologies/information that may pose a threat, including any agents subject to oversight for dual use research of concern.*

For examples of agents subject to oversight, see the United States Government Policy for Institutional Oversight of Life Sciences Dual Use Research of Concern.

Experiments of concern

Does the work involve any of these experiments of concern:

No	Yes	
☒	☐	Demonstrate how to render a vaccine ineffective
☒	☐	Confer resistance to therapeutically useful antibiotics or antiviral agents
☒	☐	Enhance the virulence of a pathogen or render a nonpathogen virulent
☒	☐	Increase transmissibility of a pathogen
☒	☐	Alter the host range of a pathogen
☒	☐	Enable evasion of diagnostic/detection modalities
☒	☐	Enable the weaponization of a biological agent or toxin
☒	☐	Any other potentially harmful combination of experiments and agents

Precautions and benefits

Biosecurity precautions — *Describe the precautions that were taken during the design and conduct of this research, or will be required in the communication and application of the research, to minimise biosecurity risks. These may include bio-containment facilities, changes to the study design/ methodology or redaction of details from the manuscript.*

Biosecurity oversight — *Describe any evaluations and oversight of biosecurity risks of this work that you have received from people or organizations outside of your immediate team.*

Benefits — *Describe the benefits that application or use of this work could bring, including benefits that may mitigate risks to public health, national security, or the health of crops, livestock or the environment.*

Communication benefits — *Describe whether the benefits of communicating this information outweigh the risks, and if so, how.*

ChIP-seq

Data deposition

☒ Confirm that both raw and final processed data have been deposited in a public database such as GEO.

☒ Confirm that you have deposited or provided access to graph files (e.g. BED files) for the called peaks.

Data access links
May remain private before publication. — All NGS data is deposited in GEO under accession number GSE163113 and GSE139309

Files in database submission — Raw Illumina fastq and bw files.

Genome browser session
(e.g. UCSC) — Not applicable.

Methodology

Replicates

All ChIP experiments were performed at least three times from different batches of cortical neurons using different antibodies and performed ChIP-seq analysis. We also sequenced input for each corresponding ChIP sample.

Sequencing depth

Samples were sequenced with around 20-50 million reads for each sample.

Antibodies

NELF-A, sc-23599 from Santa Cruz Biotech; NELF-E, ab170104 from Abcam; Pol II (N-20), sc-899X from Santa Cruz Biotech.

Peak calling parameters

ChIP peaks were called using MACS with parameters "--tsize=50 --gsize mm --nomodel True --shiftsize=65 --wig --space=10" against input chromatin samples as control data (Zhang et al., 2008). Threshold for p-value was set at 1E-9. Threshold for the fold_enrichment was set at 10. Master peaks (or reproducible peaks) were identified only if the ChIP-seq peaks overlap more than 50% of the shortest peaks in -JQ1 samples across two replicates. To find overlapping peaks among different BET proteins and to call the differentially bound peaks under different conditions, we merged the master peaks from different samples, called "merged peaks". Mergepeaks of HOMER (Heinz et al., 2010) was used.

Data quality

Sequencing reads that passed Illumina quality controls were used for alignment and only uniquely aligned reads were used for all analyses.

Software

The FASTQ reads were aligned to UCSC's mm10 genome using Bowtie2 with default parameters (Langmead and Salzberg, 2012). Reads with mapping quality less than 10 were removed using SAMtools (Li et al., 2009). To normalize the differences in sequencing depths, the mapped reads were “down sampled” to the lowest number of the uniquely mapped reads with duplicates followed by duplicate reads removal using ‘sambamba (Tarasov et al., 2015)’. The bigWig files were generated using ‘bamCoverage’ included in ‘Deeptools’ package for visualization on UCSC genome browser. The coverage values in bigWig files were normalized to RPGC (Reads per genomic content).

To generate ChIP coverage plots, we used either Ngs.plot R package (Shen et al., 2014) or HOMER. For the ngs.plot software, the parameter of Fragment (insert) length was set to 180, and Refseq database mm10 was used. For HOMER, “makeTagDirectory” program was used. We used BAM files that contained down-sampled, duplicates removed reads to create tag directories which contain tag information classified per chromosome wise. Using HOMER's in-built Perl scripts annotatePeaks.pl and analyzeRepeats.pl, data were used to create coverage plots.

Flow Cytometry

Plots

Confirm that:

- ☐ The axis labels state the marker and fluorochrome used (e.g. CD4-FITC).
- ☐ The axis scales are clearly visible. Include numbers along axes only for bottom left plot of group (a 'group' is an analysis of identical markers).
- ☐ All plots are contour plots with outliers or pseudocolor plots.
- ☐ A numerical value for number of cells or percentage (with statistics) is provided.

Methodology

Sample preparation

Describe the sample preparation, detailing the biological source of the cells and any tissue processing steps used.

Instrument

Identify the instrument used for data collection, specifying make and model number.

Software

Describe the software used to collect and analyze the flow cytometry data. For custom code that has been deposited into a community repository, provide accession details.

Cell population abundance

Describe the abundance of the relevant cell populations within post-sort fractions, providing details on the purity of the samples and how it was determined.

Gating strategy

Describe the gating strategy used for all relevant experiments, specifying the preliminary FSC/SSC gates of the starting cell population, indicating where boundaries between "positive" and "negative" staining cell populations are defined.

☐ Tick this box to confirm that a figure exemplifying the gating strategy is provided in the Supplementary Information.

Magnetic resonance imaging

Experimental design

Design type

Indicate task or resting state; event-related or block design.

Design specifications

Specify the number of blocks, trials or experimental units per session and/or subject, and specify the length of each trial or block (if trials are blocked) and interval between trials.

Behavioral performance measures | *State number and/or type of variables recorded (e.g. correct button press, response time) and what statistics were used to establish that the subjects were performing the task as expected (e.g. mean, range, and/or standard deviation across subjects).*

Acquisition

Imaging type(s) | *Specify: functional, structural, diffusion, perfusion.*

Field strength | *Specify in Tesla*

Sequence & imaging parameters | *Specify the pulse sequence type (gradient echo, spin echo, etc.), imaging type (EPI, spiral, etc.), field of view, matrix size, slice thickness, orientation and TE/TR/flip angle.*

Area of acquisition | *State whether a whole brain scan was used OR define the area of acquisition, describing how the region was determined.*

Diffusion MRI ☐ Used ☐ Not used

Preprocessing

Preprocessing software | *Provide detail on software version and revision number and on specific parameters (model/functions, brain extraction, segmentation, smoothing kernel size, etc.).*

Normalization | *If data were normalized/standardized, describe the approach(es): specify linear or non-linear and define image types used for transformation OR indicate that data were not normalized and explain rationale for lack of normalization.*

Normalization template | *Describe the template used for normalization/transformation, specifying subject space or group standardized space (e.g. original Talairach, MNI305, ICBM152) OR indicate that the data were not normalized.*

Noise and artifact removal | *Describe your procedure(s) for artifact and structured noise removal, specifying motion parameters, tissue signals and physiological signals (heart rate, respiration).*

Volume censoring | *Define your software and/or method and criteria for volume censoring, and state the extent of such censoring.*

Statistical modeling & inference

Model type and settings | *Specify type (mass univariate, multivariate, RSA, predictive, etc.) and describe essential details of the model at the first and second levels (e.g. fixed, random or mixed effects; drift or auto-correlation).*

Effect(s) tested | *Define precise effect in terms of the task or stimulus conditions instead of psychological concepts and indicate whether ANOVA or factorial designs were used.*

Specify type of analysis: ☐ Whole brain ☐ ROI-based ☐ Both

Statistic type for inference (See Eklund et al. 2016) | *Specify voxel-wise or cluster-wise and report all relevant parameters for cluster-wise methods.*

Correction | *Describe the type of correction and how it is obtained for multiple comparisons (e.g. FWE, FDR, permutation or Monte Carlo).*

Models & analysis

n/a	Involved in the study
☐	☐ Functional and/or effective connectivity
☐	☐ Graph analysis
☐	☐ Multivariate modeling or predictive analysis

Functional and/or effective connectivity | *Report the measures of dependence used and the model details (e.g. Pearson correlation, partial correlation, mutual information).*

Graph analysis | *Report the dependent variable and connectivity measure, specifying weighted graph or binarized graph, subject- or group-level, and the global and/or node summaries used (e.g. clustering coefficient, efficiency, etc.).*

Multivariate modeling and predictive analysis | *Specify independent variables, features extraction and dimension reduction, model, training and evaluation metrics.*
