# Supplementary material for: Enhancer RNAs stimulate Pol II pause release by harnessing multivalent interactions to NELF
Source: Nat Commun. 2022 May 4;13:2429. doi: 10.1038/s41467-022-29934-w (PMC9068813; doi:10.1038/s41467-022-29934-w)

Source Data Fig. 2b

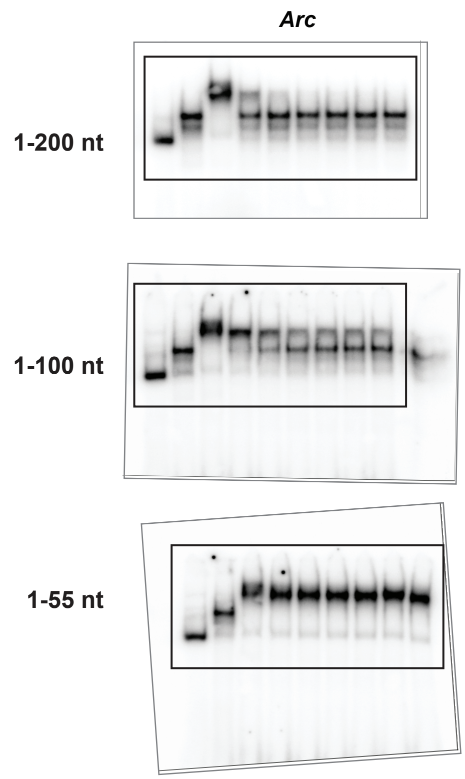

Source Data Fig. 2c

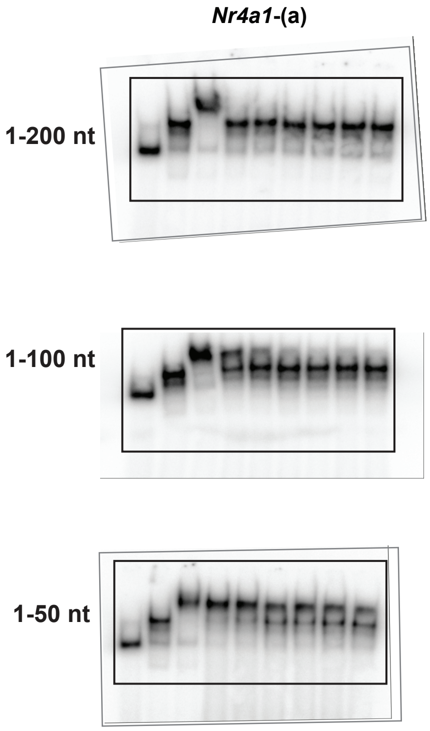

Source Data Fig. 2d

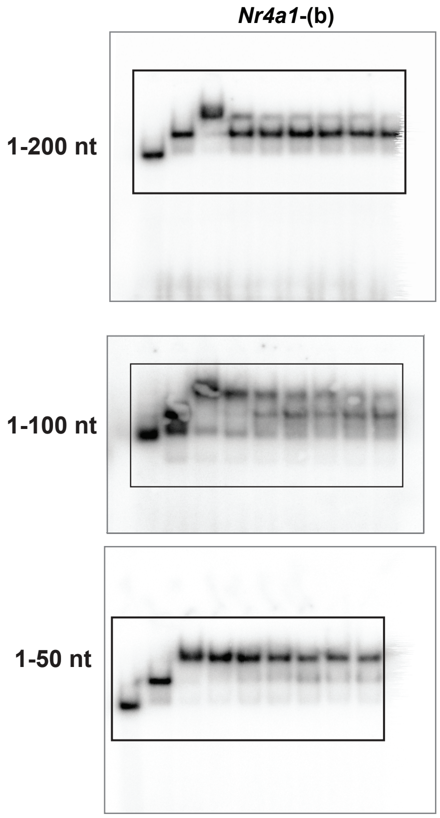

Source Data Fig. 2f

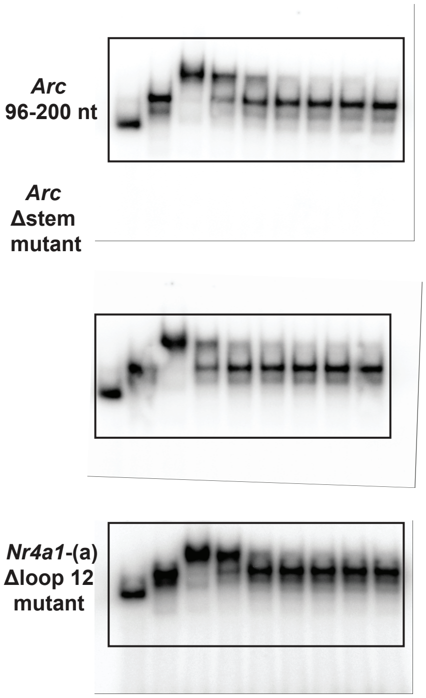

Source Data Fig. 2g

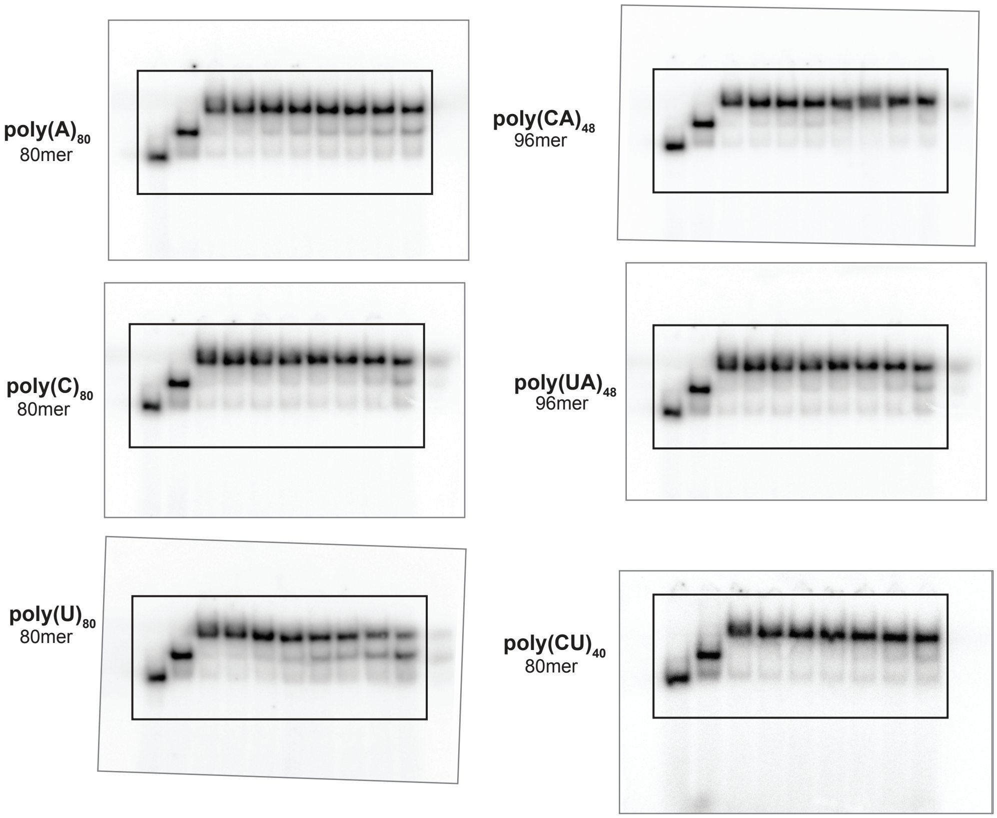

Source Data Fig. 2h

poly(GU)$_{40}$
80mer

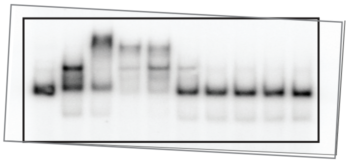

poly(GA)$_{48}$
96mer

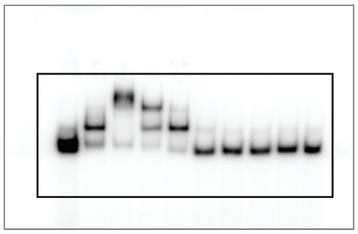

poly(GA)$_{20}$
40mer

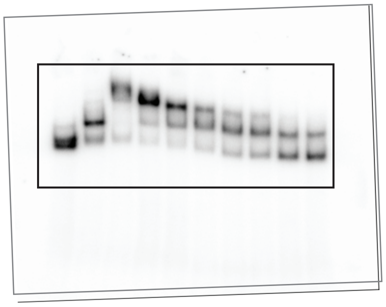

Source Data Fig. 2i

poly(G$_2$A)
96mer

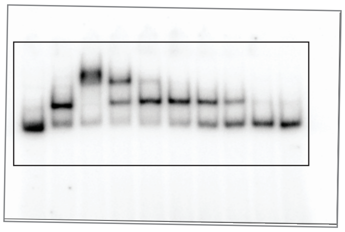

poly(G$_2$A$_3$)
96mer

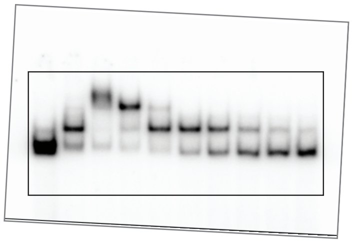

poly(G$_2$A$_6$)
96mer

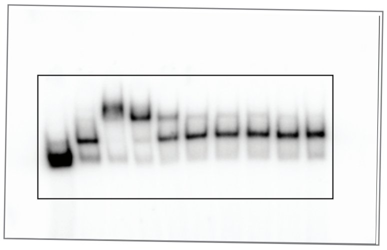

Source Data Fig. 3a

Arc

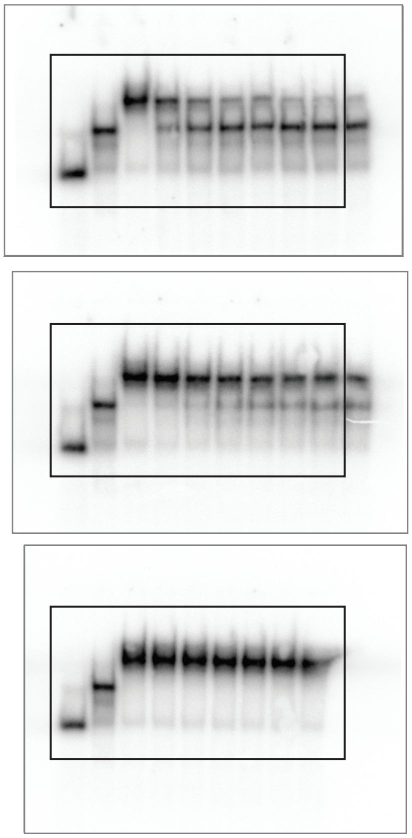

Source Data Fig. 3b

Arc

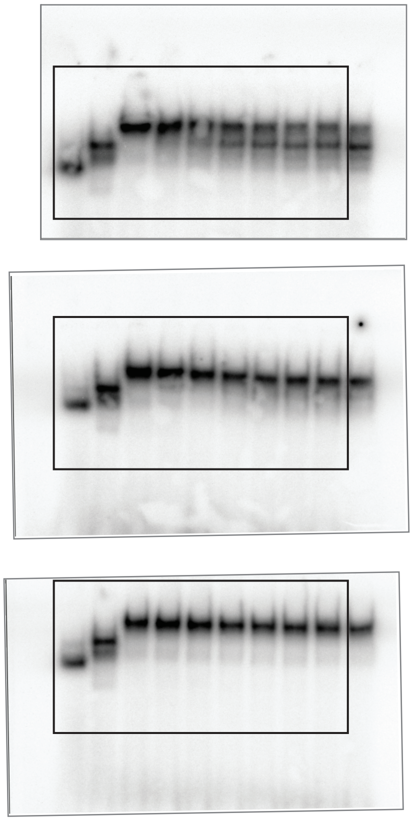

Source Data Fig. 3d

Arc

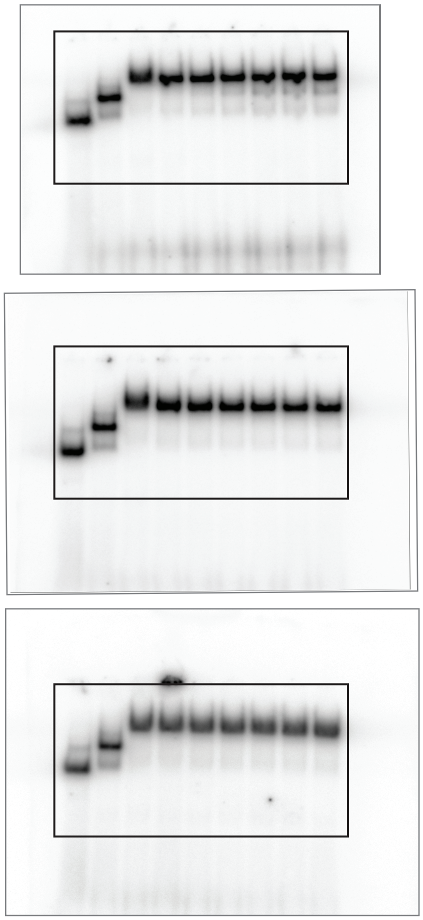

Source Data Fig. 3e

***Nr4a1*-(a)**

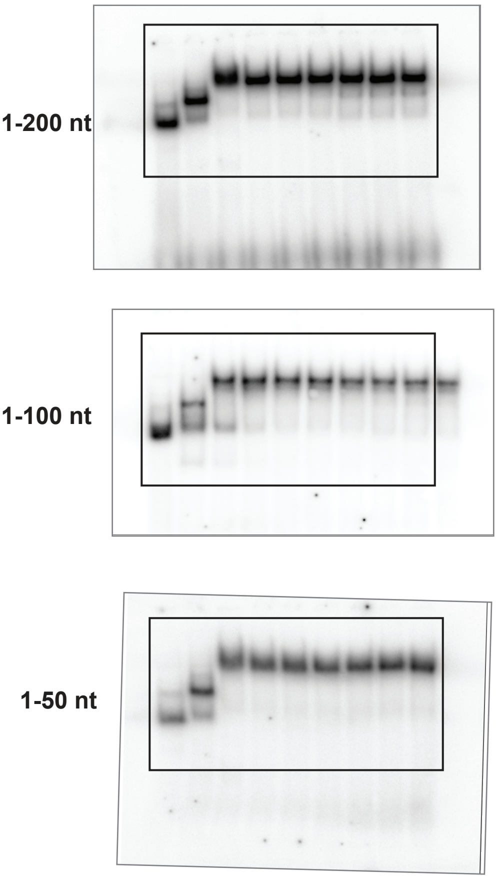

Source Data Fig. 3f

***Nr4a1*-(b)**

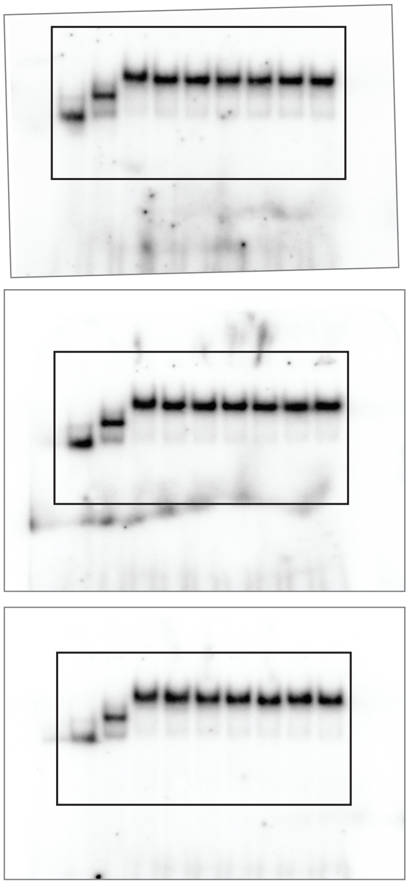

Source Data Fig. 4a

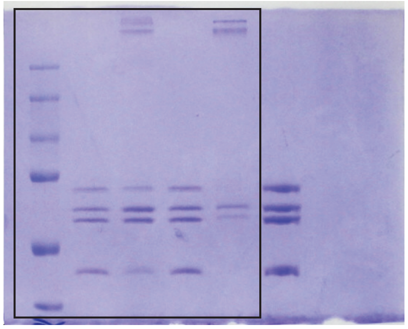

Source Data Fig. 6b

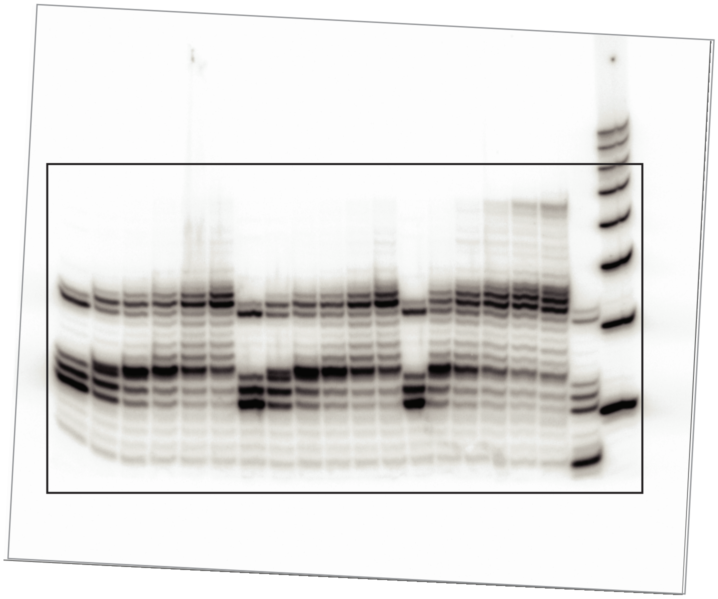

Source Data Fig. 6c

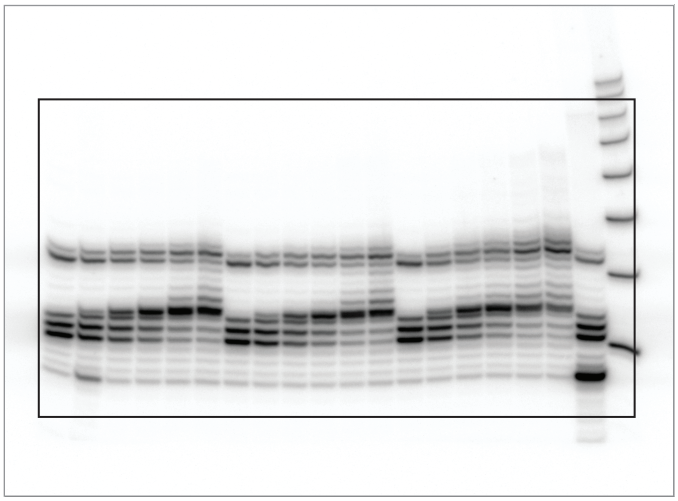

Source Data Fig. 6d

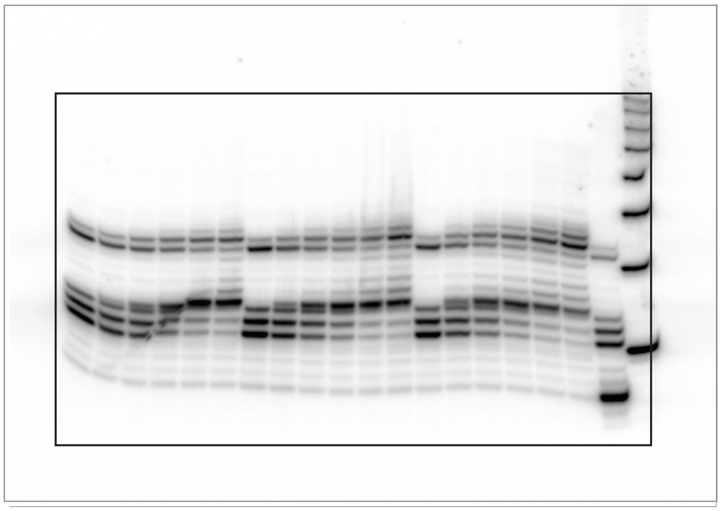

Source Data Supplementary Fig. 1a

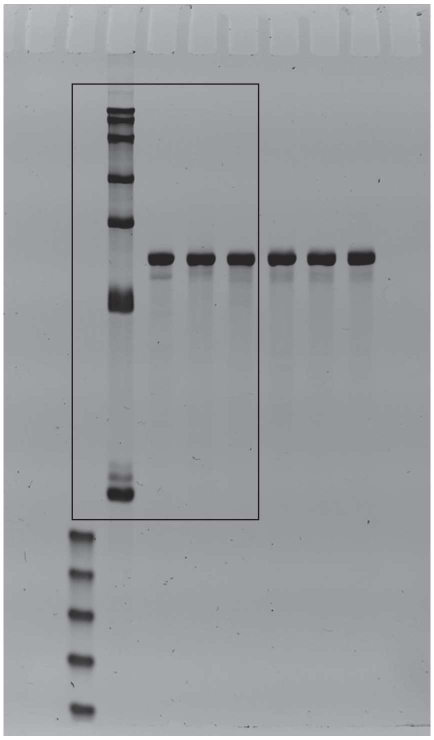

Source Data Supplementary Fig. 2b

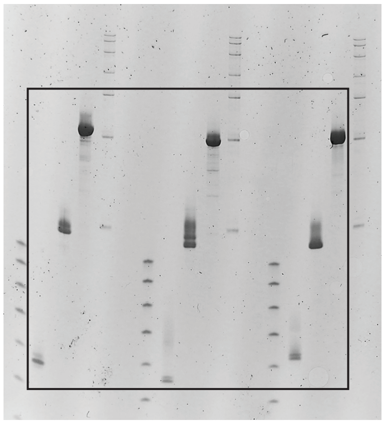

Source Data Supplementary Fig. 2c

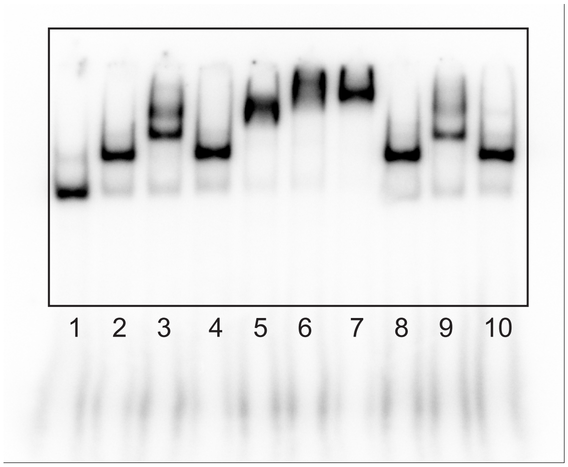

lane 3,6,9 anti-SPT5 (D-3) X (Santa Cruz Biotech; Cat# sc-133217X)
lane 4,7,10 anti-NELF-E (Abcam; Cat# ab170104)

Source Data Supplementary Fig. 2d

Arc

1-200 nt

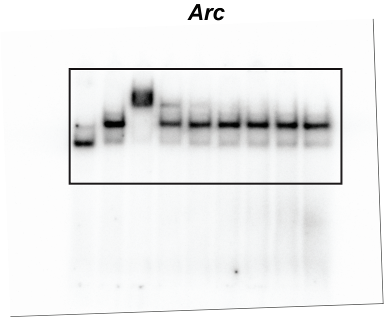

1-100 nt

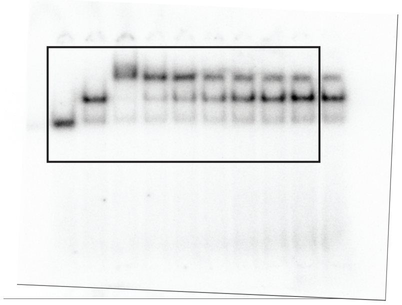

1-55 nt

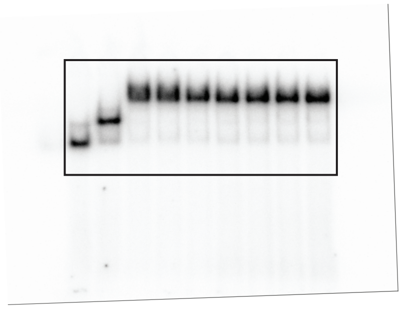

Source Data Supplementary Fig. 2g

***Nr4a1*-(a)**

G-less G-to-A 1-50 nt

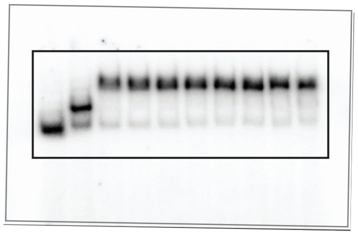

G-less G-to-A 1-100 nt

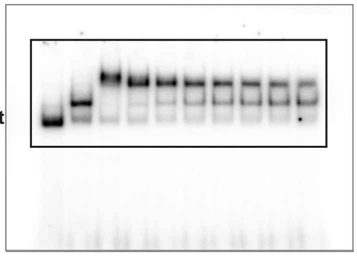

G-less G-to-C 1-100 nt

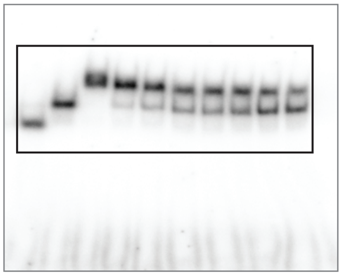

Source Data Supplementary Fig. 2i

G-reduced 2G middle

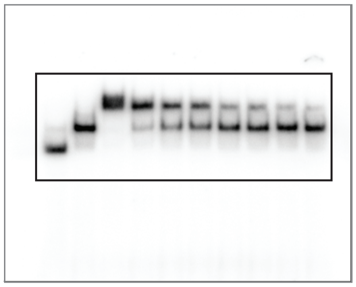

G-reduced 3G at 3'end

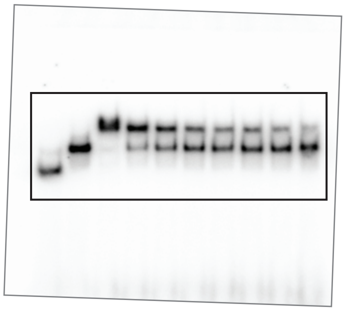

G-reduced 6G at 3'end

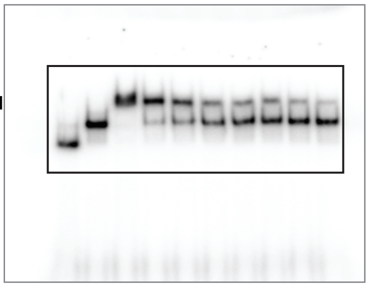

Source Data Supplementary Fig. 3a

NELF variants titration

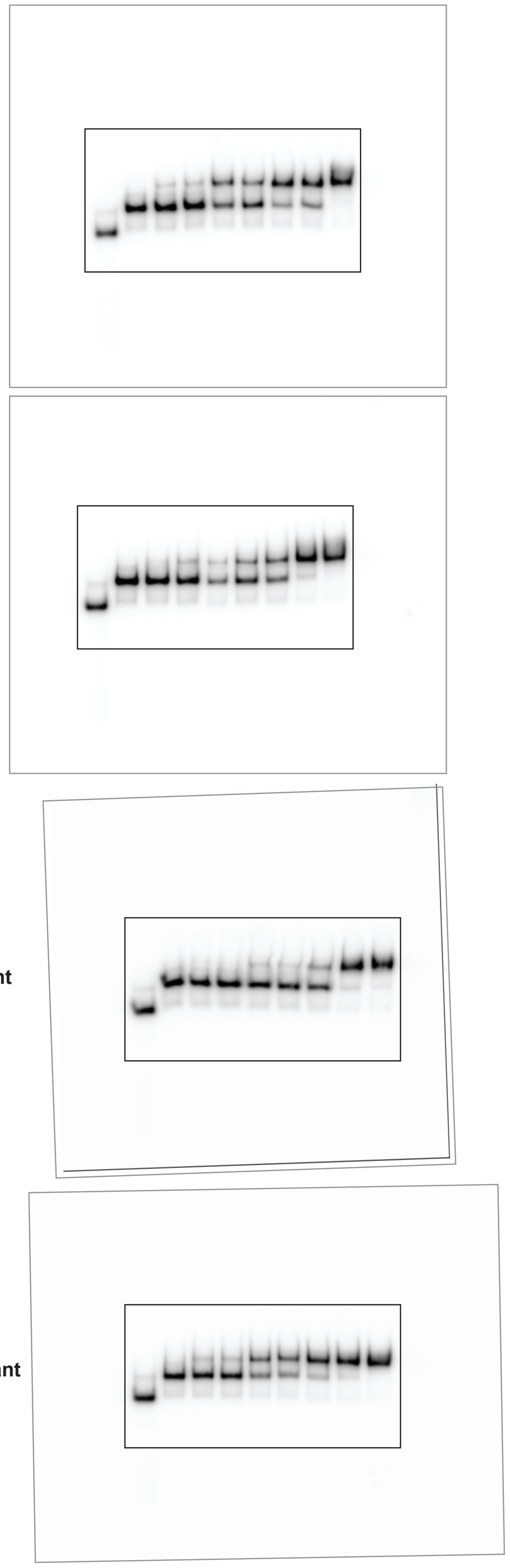

NELF WT

NELF ΔRRM

NELF-C patch mutant

NELF double mutant

Source Data Supplementary Fig. 3b

NELFΔRRM

***Nr4a1*-(a)** ***Nr4a1*-(b)**

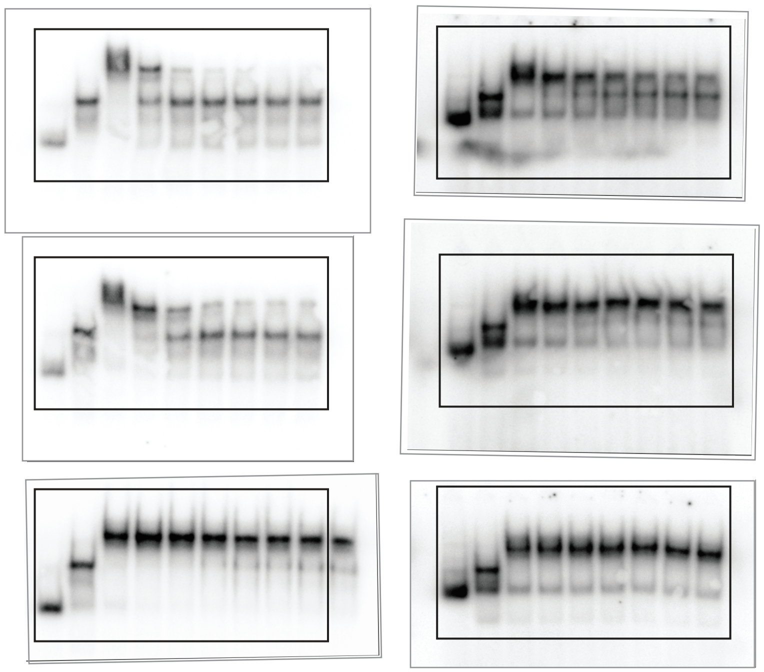

1-200 nt

1-100 nt

1-50 nt

Source Data Supplementary Fig. 3c

NELF-C patch mutant

***Nr4a1*-(a)** ***Nr4a1*-(b)**

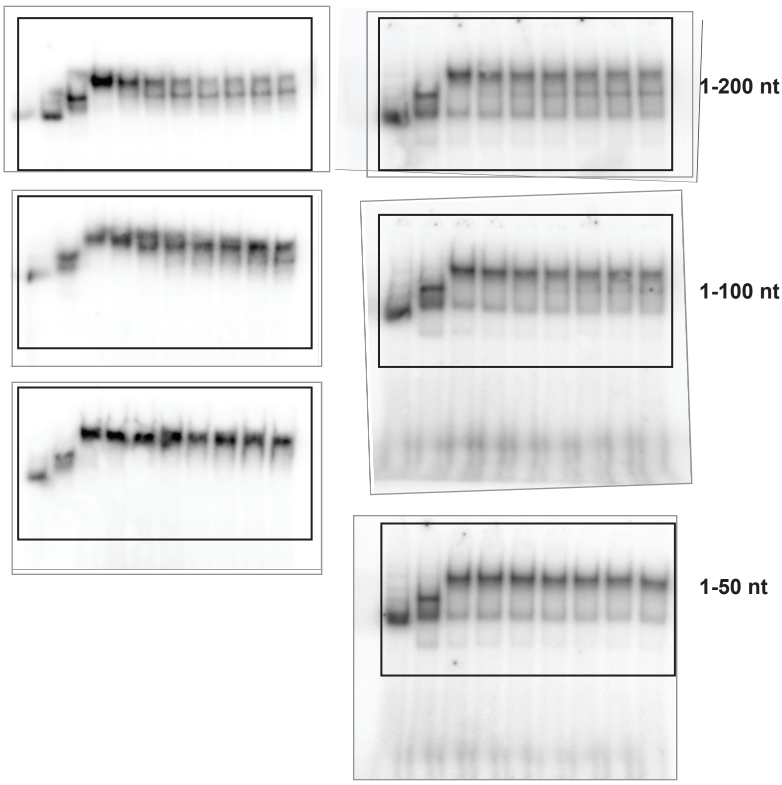

1-200 nt

1-100 nt

1-50 nt

1-200 nt

1-100 nt

1-50 nt

Source Data Supplementary Fig. 4a

Urea-PAGE
NELF + eRNA

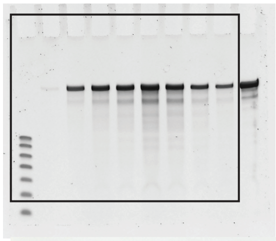

SDS-PAGE
NELF + eRNA

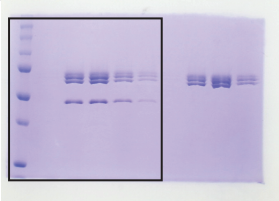

SDS-PAGE
NELF only

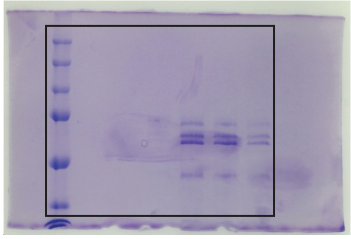

Urea-PAGE
eRNA only

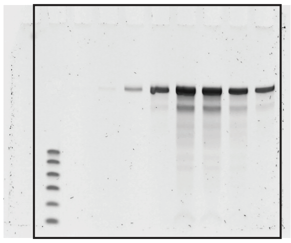

Source Data Supplementary Fig. 4b

SDS-PAGE
NELF double mutant
+ eRNA

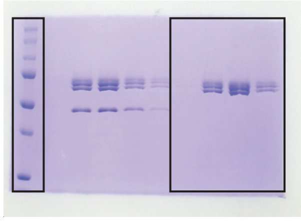

SDS-PAGE
NELF double mutant
only

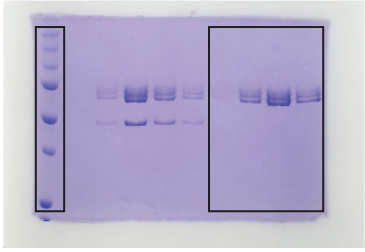

Source Data Supplementary Fig. 4e

+ unlabeled (0% 4SU) ***Nr4a1*-(a) eRNA 1-100** | **+ 40% 4SU-labeled** ***Nr4a1*-(a) eRNA 1-100**

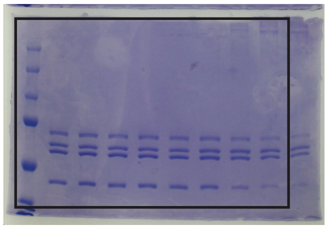

NELF protein only | **+ 80% 4SU-labeled** ***Nr4a1*-(a) eRNA 1-100**

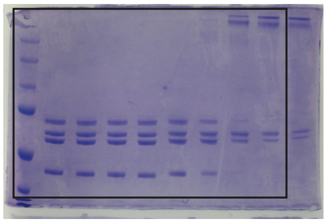

***Nr4a1*-(a) eRNA 1-100**

RNA only | **+ NELF**

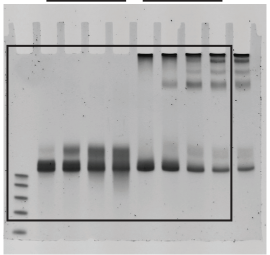

40% 4SU

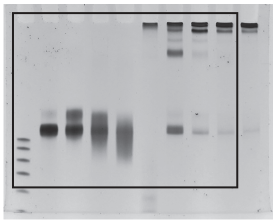

80% 4SU

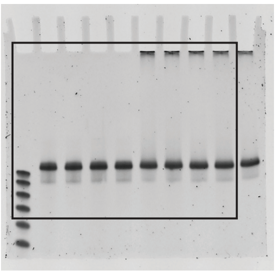

unlabeled
(0% 4SU)

Source Data Supplementary Fig. 4f

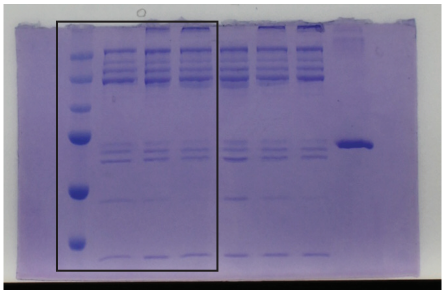

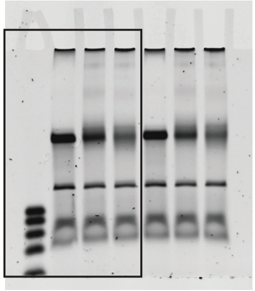

Source Data Supplementary Fig. 7b

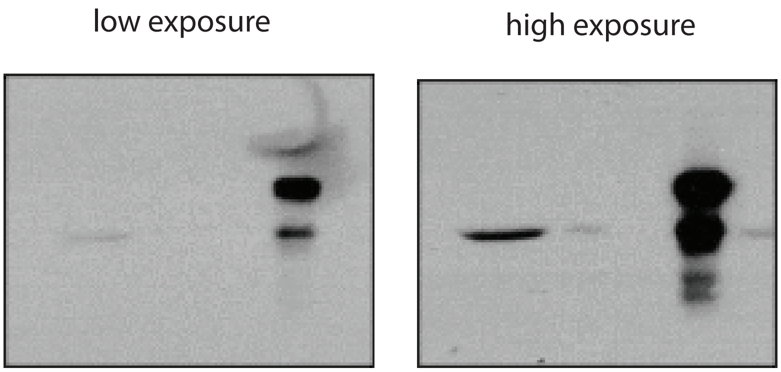

anti-NELF-E
(Abcam; Cat# ab170104)

Source Data Supplementary Fig. 10a

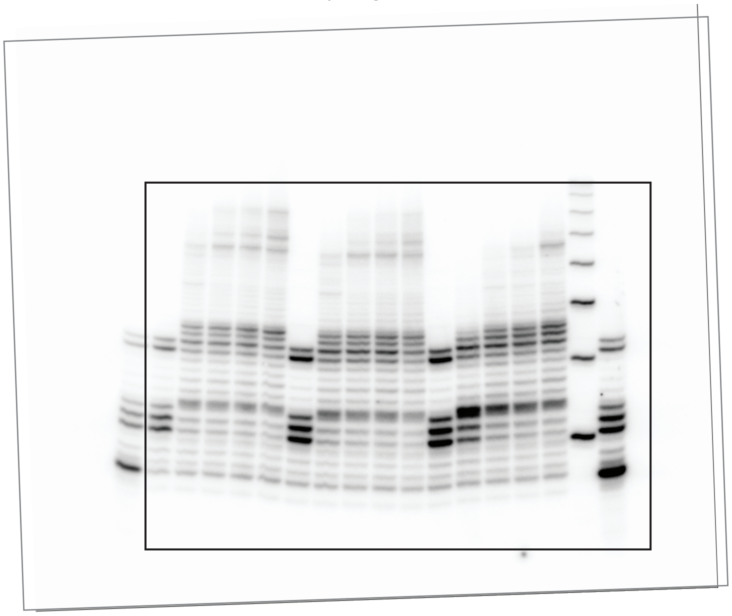

Source Data Supplementary Fig. 10b

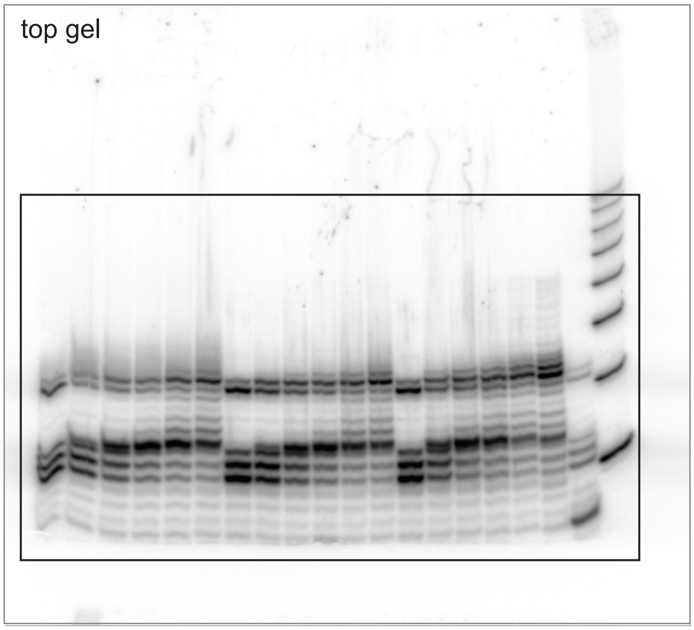

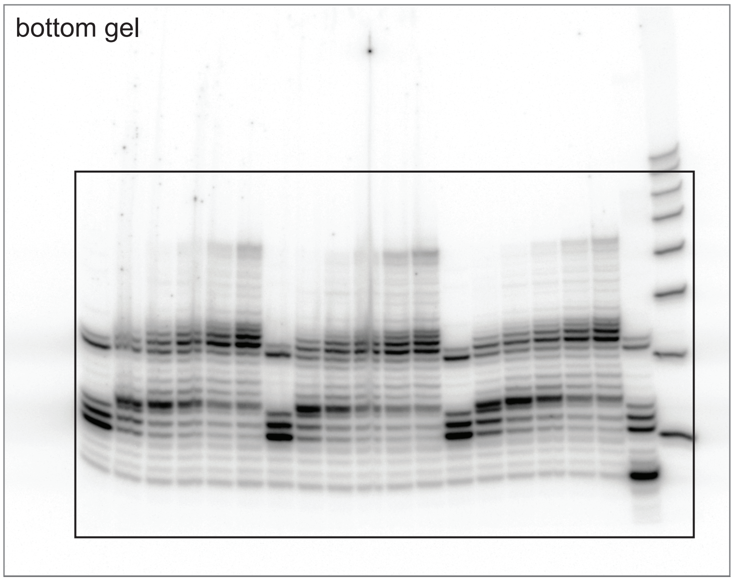

Source Data Supplementary Fig. 10c

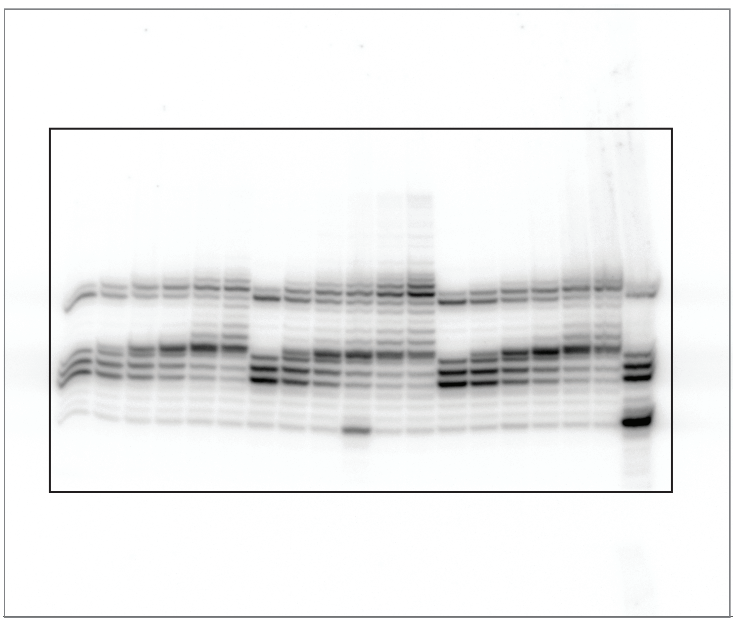

Source Data Supplementary Fig. 10d

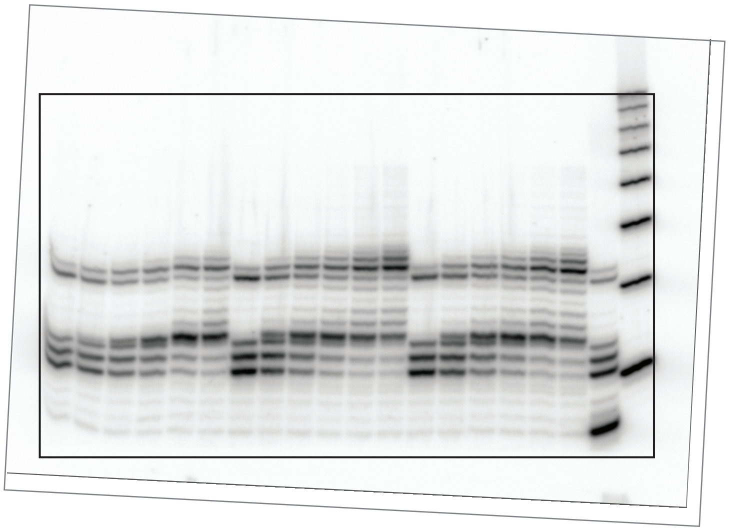

Supplement: Supplementary file 12 — Source Data [file 41467_2022_29934_MOESM12_ESM.zip › Source Data/Source_Data_uncropped_gels.pdf]
